# Supplementary material for: Conjugates of Chloramphenicol Amine and Berberine as Antimicrobial Agents
Source: Antibiotics (Basel). 2022 Dec 22;12(1):15. doi: 10.3390/antibiotics12010015 (PMC9854996; doi:10.3390/antibiotics12010015)
Supplement: Supplementary file 1 [file antibiotics-12-00015-s001.zip › antibiotics-2116858-supplementary.pdf]

Supplementary Materials

FOR THE ARTICLE

Conjugates of Chloramphenicol Amine and Berberine as Antimicrobial Agents

Julia A. Pavlova[1,2,†], Andrey G. Tereshchenkov[3,†], Pavel A. Nazarov[3,*], Dmitrii A. Lukianov[1,2], Dmitry A. Skvortsov[1], Vladimir I. Polshakov[4], Byasilya F. Vasilieva[5], Olga V. Efremenkova[5], Mikhail Y. Kaiumov[6], Alena Paleskava[6,7], Andrey L. Konevega[6,7,8], Olga A. Dontsova[1,2,9], Ilya A. Osterman[1,2], Alexey A. Bogdanov[1,3], and Natalia V. Sumbatyan[1,*]

[1] Lomonosov Moscow State University, Department of Chemistry, 1/3 Leninskie Gory, 119991 Moscow, Russia; julidev@yandex.ru (J.A.P.); dmitrii.lukianov@skoltech.ru (D.A.L.); skvorratd@mail.ru (D.A.S.); dontsova@belozersky.msu.ru (O.A.D.); i.osterman@skoltech.ru (I.A.O.); bogdanov@belozersky.msu.ru (A.A.B.)

[2] Center of Life Sciences, Skolkovo Institute of Science and Technology, 143028 Skolkovo, Russia

[3] Lomonosov Moscow State University, A.N. Belozersky Institute of Physico-Chemical Biology, 1/40 Leninskie Gory, 119992 Moscow, Russia; tereshchenkov@list.ru

[4] Lomonosov Moscow State University, Faculty of Fundamental Medicine, 27/1 Lomonosvsky Ave., 119991 Moscow, Russia; vpolsha@mail.ru

[5] Gause Institute of New Antibiotics, 11 B. Pirogovskaya Street, 119021 Moscow, Russia; instna@sovintel.ru (B.F.V.); ovefr@yandex.ru (O.V.E.)

[6] Petersburg Nuclear Physics Institute, NRC "Kurchatov Institute", 188300 Gatchina, Russia; kaiumov_my@pnpi.nrcki.ru (M.Y.K.); polesskova_ev@pnpi.nrcki.ru (A.P.); konevega_al@pnpi.nrcki.ru (A.L.K.)

[7] Institute of Biomedical Systems and Biotechnologies, Peter the Great St.Petersburg Polytechnic University, 195251 Saint Petersburg, Russia

[8] NRC "Kurchatov Institute", 123182 Moscow, Russia

[9] Shemyakin-Ovchinnikov Institute of Bioorganic Chemistry, 117997 Moscow, Russia

† The authors contributed equally to this work.

* Correspondence: sumbtyan@belozersky.msu.ru (N.V.S.); nazarovpa@gmail.com (P.A.N.)

This file includes:

I. Supplementary Methods

II. Supplementary Table S1

III. Supplementary Figures (S1–S16 with legends)

I. SUPPLEMENTARY METHODS

Detailed Synthesis of CAM-C1-BER

N-[(1R,2R)-1,3-dihydroxy-1-(4-nitrophenyl)propan-2-yl]-2-(2-[(13RS,13aRS)-9,10-dimethoxy-5,8,13,13a-tetrahydro-6H-benzo[g]-1,3-benzodioxolo[5,6-a]quinolizin-13-yl]acetamido)acetamide (CAM-C1-thBER)

CAM-C1-thBER was obtained as CAM-C8-thBER from 75 mg (0.173 mmol) of thBER-CH_2-COOH·HCl, 28 mg (0.245 mmol) of HOSu, 50.5 mg (0.245 mmol) of DCC, 66 μL (0.378 mmol) of DIPEA, and 72 mg (0.189 mmol) of CAM-C1-NH_2·TFA. The target product was

isolated on a silica gel column eluting with a solvent system of CH_2Cl_2:MeOH = 9:1. As a result, a light yellow solid was obtained. Yield: 82 mg (73%); TLC: R_f (CH_2Cl_2:MeOH, 9:1) 0.45; LC-MS *m/z* calculated for $C_{33}H_{37}N_4O_{10}$ $(M+H)^+$: 649.25, found 648.95; t_R = 1.34 min; ^{1}H NMR (DMSO-d_6, 400 MHz) δ (ppm) 8.11 & 8.09 (2H, d, *J* = 8.6 Hz, *o*-H NO_2-Phe), 7.81 & 7.74 (1H, t, *J* = 5.8 Hz, -CH_2-NH-CO-), 7.54 & 7.52 (2H, d, *J* = 8.6 Hz, *m*-H NO_2-Phe), 7.37 & 7.36 (1H, d, *J* = 9.1 Hz, -CH-NH-CO-), 6.87 & 6.80 (1H, d, *J* = 8.5 Hz, H-12), 6.84 (1H, s, H-1), 6.82 & 6.77 (1H, d, *J* = 8.5 Hz, H-11), 6.67 (1H, s, H-4), 5.96 & 5.93 (1H, d, *J* = 1.1 Hz, O-CH_2^a-O), 5.95 & 5.92 (1H, d, *J* = 1.1 Hz, O-CH_2^b-O), 5.86 & 5.83 (1H, d, *J* = 4.5 Hz, -CH-OH), 5.00 & 4.99 (1H, t, *J* = 4.5 Hz, -CH-OH), 4.87 & 4.86 (1H, dd, *J* = 5.9, 2.0 Hz, -CH_2-OH), 4.07 & 4.06 (1H, d, *J* = 16.0 Hz, H-8^a), 3.96–3.84 (1H, m, -CH-NH-CO-), 3.76 & 3.75 (3H, s, C_{10}-OCH_3), 3.72 & 3.71 (3H, s, C_9-OCH_3), 3.66–3.46 (4H, m, H-13, H-13a, -CH_2^a-OH, -CH_2^a-NH-), 3.44–3.38 (1H, m, H-8^b), 3.35–3.23 (2H, m, -CH_2^b-OH, -CH_2^b-NH-), 3.06 (1H, br d, *J* = 10.5 Hz, H-6^a), 2.93–2.82 (1H, m, H-5^a), 2.56 (1H, d, *J* = 15.4 Hz, H-5^b), 2.41 (1H, t, *J* = 11.4 Hz, H-6^b), 2.23–2.12 (1H, m, C_{13}-CH_2^a-CO), 1.94–1.84 (1H, m, C_{13}-CH_2^b-CO); ^{13}C NMR (DMSO-d_6, 101 MHz) δ (ppm) 172.03 & 171.84 (-CH-NH-CO-), 168.88 & 168.75 (-CH_2-NH-CO-), 151.86 & 151.82 (NO_2-Ph_{para}), 150.10 & 150.06 (C-10), 146.36 & 146.33 (NO_2-Ph_{ipso}), 145.99 & 145.97 (C-2), 145.53 & 145.50 (C-3), 144.33 & 144.30 (C-9), 132.35 & 132.31 (C-12a), 129.25 (C-4a), 128.70 & 128.69 (C-13b), 127.56 & 127.52 (C-8a), 127.33 (2C, NO_2-Ph_{meta}), 124.01 (C-12), 122.85 (2C, NO_2-Ph_{ortho}), 110.97 & 110.89 (C-11), 108.07 (C-4), 105.86 & 105.82 (C-1), 100.65 (O-CH_2-O), 69.07 & 68.99 (-CH-OH), 62.75 (C-13a), 60.40 & 60.36 (-CH_2-OH), 59.46 & 59.45 (C_9-OCH_3), 56.09 & 56.02 (-NH-CH-), 55.64 & 55.61 (C_{10}-OCH_3), 53.85 (C-8), 50.56 (C-6), 42.22 & 41.92 (-CH_2-NH-), 39.24 (C-13), 38.81 & 38.77 (-NH-CO-CH_2-C_{13}), 29.13 (C-5).

13-{[({[(1R,2R)-1,3-dihydroxy-1-(4-nitrophenyl)propan-2-yl]carbamoyl}methyl)carbamoyl]methyl}-9,10-dimethoxy-5,6-dihydrobenzo[g]-1,3-benzodioxolo[5,6-a]quinolizinium hydroxide (CAM-C1-BER)

CAM-C1-BER was obtained as CAM-C8-BER from 70 mg (0.108 mmol) of CAM-C1-thBER and 38 mg (0.216 mmol) of NBS. The target product was isolated on a silica gel column eluting with a solvent system of $CHCl_3$:MeOH:NH_4OH = 65:25:4. As a result, a yellow solid was obtained. Yield: 18.8 mg (26%); TLC: R_f ($CHCl_3$:MeOH:NH_4OH, 65:25:4) 0.46; LC-MS *m/z* calculated for $C_{33}H_{33}N_4O_{10}$ $(M)^+$: 645.22, found 645.15; t_R = 1.24 min; ^{1}H NMR (DMSO-d_6, 400 MHz) δ (ppm) 9.96 (1H, s, H-8), 8.84 (1H, t, *J* = 5.7 Hz, -CH_2-NH-CO-), 8.12 (1H, d, *J* = 9.3 Hz, H-11), 8.10 (2H, d, *J* = 8.8 Hz, *o*-H NO_2-Phe), 7.96 (1H, d, *J* = 9.3 Hz, H-12), 7.83 (1H, d, *J* = 9.2 Hz, -CH-NH-CO-), 7.61 (2H, d, *J* = 8.8 Hz, *m*-H NO_2-Phe), 7.60 (1H, s, H-1), 7.14 (1H, s, H-4), 6.14 (2H, s, O-CH_2-O), 5.05 (1H, d, *J* = 2.5 Hz, -CH-OH), 4.84 (2H, br s, H-6), 4.23 (2H, s, C_{13}-CH_2-CONH), 4.09 (3H, s, C_9-OCH_3), 4.06 (3H, s, C_{10}-OCH_3), 4.05–3.99 (1H, m, -CH-NH-CO-), 3.86 (1H, dd, *J* = 16.8, 5.7 Hz, -CH_2^a-NH-), 3.73 (1H, dd, *J* = 16.8, 5.7 Hz, -CH_2^b-NH-), 3.59 (1H, dd, *J* = 10.5, 7.9 Hz, -CH_2^a-OH), 3.33 (1H, dd, *J* = 10.5, 5.9 Hz, -CH_2^b-OH), 3.09 (2H, t, *J* = 5.9 Hz, H-5); ^{13}C NMR (DMSO-d_6, 101 MHz) δ (ppm) 169.82 (-CH-NH-CO-), 168.33 (-CH_2-NH-CO-), 151.92 (NO_2-Ph_{para}), 150.31 (C-10), 149.35 (C-3), 146.71 (C-2), 146.26 (NO_2-Ph_{ipso}), 145.25 (C-8), 144.23 (C-9), 137.44 (C-13a), 134.01 (C-4a), 133.05 (C-12a), 127.79 (C-13), 127.47 (2C, NO_2-Ph_{meta}), 126.15 (C-11), 122.84 (2C, NO_2-Ph_{ortho}), 120.92 (C-8a), 120.91 (C-12), 120.11 (C-13b), 109.27 (C-1), 108.36 (C-4), 102.05 (O-CH_2-O), 69.31 (-CH-OH), 62.08 (C_9-OCH_3), 60.49 (-CH_2-OH), 57.03 (C_{10}-OCH_3), 56.88 (C-6), 56.17 (-NH-CH-), 42.07 (-CH_2-NH-), 37.33 (-NH-CO-CH_2-C_{13}), 27.27 (C-5).

Detailed Synthesis of CAM-C2-BER

N-[(1R,2R)-1,3-dihydroxy-1-(4-nitrophenyl)propan-2-yl]-3-(2-[(13RS,13aRS)-9,10-dimethoxy-5,8,13,13a-tetrahydro-6H-benzo[g]-1,3-benzodioxolo[5,6-a]quinolizin-13-yl]acetamido)propanamide (CAM-C2-thBER)

CAM-C2-thBER was obtained as CAM-C8-thBER from 75 mg (0.173 mmol) of thBER-CH_2-COOH·HCl, 28 mg (0.245 mmol) of HOSu, 50.5 mg (0.245 mmol) of DCC, 66 μL (0.378 mmol) of DIPEA, and 75 mg (0.189 mmol) of CAM-C2-NH_2·TFA. The target product was isolated on a silica gel column eluting with a solvent system of CH_2Cl_2:MeOH = 9:1. As a result, a light yellow solid was obtained. Yield: 74 mg (65%); TLC: R_f (CH_2Cl_2:MeOH, 9:1) 0.36; LC-MS *m/z* calculated for $C_{34}H_{39}N_4O_{10}$ $(M+H)^+$: 663.27, found 662.86; t_R = 1.36 min; ^1H NMR (DMSO-d_6, 400 MHz) δ (ppm) 8.113 & 8.108 (2H, d, *J* = 8.6 Hz, *o*-H NO_2-Phe), 7.54 (1H, d, *J* = 9.4 Hz, -CH-N$\underline{H}$-CO-), 7.53 (2H, d, *J* = 8.6 Hz, *m*-H NO_2-Phe), 7.48 (1H, t, *J* = 5.7 Hz, -CH_2-N$\underline{H}$-CO-), 6.88–6.80 (3H, m, H-12, H-11, H-1), 6.66 (1H, s, H-4), 5.95 (1H, d, *J* = 2.4 Hz, O-C$\underline{H}_2^a$-O), 5.94 (1H, d, *J* = 2.4 Hz, O-C$\underline{H}_2^b$-O), 5.79 & 5.78 (1H, d, *J* = 4.5 Hz, -CH-O$\underline{H}$), 4.98 (1H, t, *J* = 3.7 Hz, -C$\underline{H}$-OH), 4.83–4.77 (1H, m, -CH_2-O$\underline{H}$), 4.06 (1H, d, *J* = 15.9 Hz, H-8^a), 3.95 (1H, q, *J* = 7.3 Hz, -C$\underline{H}$-NH-CO-), 3.77 (3H, s, C_{10}-OC$\underline{H}_3$), 3.72 (3H, s, C_9-OC$\underline{H}_3$), 3.63–3.57 (2H, m, H-13, H-13a), 3.51 (1H, dt, *J* = 9.7, 7.0 Hz, -C$\underline{H}_2^a$-OH), 3.40 (1H, d, *J* = 15.9 Hz, H-8^b), 3.25 (1H, tt, *J* = 10.5, 5.3 Hz, -C$\underline{H}_2^b$-OH), 3.06 (1H, dd, *J* = 10.9, 4.5 Hz, H-6^a), 3.01–2.81 (3H, m, β-C$\underline{H}_2$, H-5^a), 2.57 (1H, d, *J* = 14.4 Hz, H-5^b), 2.41 (1H, td, *J* = 10.9, 2.8 Hz, H-6^b), 2.10 (1H, d, *J* = 14.3 Hz, C_{13}-C$\underline{H}_2^a$-CO), 2.08–1.93 (2H, m, *α*-C$\underline{H}_2$), 1.81 (1H, dd, *J* = 14.3, 2.5 Hz, C_{13}-C$\underline{H}_2^b$-CO); ^{13}C NMR (DMSO-d_6, 101 MHz) δ (ppm) 171.47 & 171.44 (-CH-NH-$\underline{C}$O-), 170.26 & 170.25 (-CH_2-NH-$\underline{C}$O-), 151.95 & 151.93 (NO_2-Ph_{para}), 150.09 (C-10), 146.32 (NO_2-Ph_{ipso}), 145.93 (C-2), 145.46 (C-3), 144.31 (C-9), 132.28 & 132.26 (C-12a), 129.21 (C-4a), 128.71 (C-13b), 127.58 (C-8a), 127.39 & 127.37 (2C, NO_2-Ph_{meta}), 124.05 & 124.04 (C-12), 122.77 (2C, NO_2-Ph_{ortho}), 110.90 (C-11), 108.05 (C-4), 105.84 (C-1), 100.64 (O-$\underline{C}H_2$-O), 69.49 & 69.45 (-$\underline{C}$H-OH), 62.73 (C-13a), 60.47 & 60.44 (-$\underline{C}H_2$-OH), 59.45 (C_9-O$\underline{C}H_3$), 55.86 (-NH-$\underline{C}$H-), 55.66 (C_{10}-O$\underline{C}H_3$), 53.89 (C-8), 50.58 (C-6), 39.46 (C-13), 38.85 (-NH-CO-$\underline{C}H_2$-C_{13}), 35.41 (β-CH_2), 35.25 & 35.22 (*α*-CH_2), 29.13 (C-5).

13-{[(2-{[(1R,2R)-1,3-dihydroxy-1-(4-nitrophenyl)propan-2-yl]carbamoyl}ethyl)carbamoyl]methyl}-9,10-dimethoxy-5,6-dihydrobenzo[g]-1,3-benzodioxolo[5,6-a]quinolizinium hydroxide (CAM-C2-BER)

CAM-C2-BER was obtained as CAM-C8-BER from 45 mg (0.068 mmol) of CAM-C2-thBER and 24 mg (0.136 mmol) of NBS. The target product was isolated on a silica gel column eluting with a solvent system of $CHCl_3$:MeOH:NH_4OH = 65:25:4. As a result, a yellow-brown solid was obtained. Yield: 20 mg (43%); TLC: R_f ($CHCl_3$:MeOH:NH_4OH, 65:25:4) 0.48; LC-MS *m/z* calculated for $C_{34}H_{35}N_4O_{10}$ $(M)^+$: 659.23, found 659.24; t_R = 1.31 min; ^1H NMR (DMSO-d_6, 400 MHz) δ (ppm) 9.96 (1H, s, H-8), 8.72 (1H, t, *J* = 5.7 Hz, -CH_2-N$\underline{H}$-CO-), 8.17 (1H, d, *J* = 9.4 Hz, H-11), 8.10 (2H, d, *J* = 8.5 Hz, *o*-H NO_2-Phe), 7.90 (1H, d, *J* = 9.4 Hz, H-12), 7.77 (1H, d, *J* = 9.1 Hz, -CH-N$\underline{H}$-CO-), 7.61 (1H, s, H-1), 7.60 (2H, d, *J* = 8.5 Hz, *m*-H NO_2-Phe), 7.16 (1H, s, H-4), 6.16 (2H, d, *J* = 3.5 Hz, O-C$\underline{H}_2$-O), 5.86 (1H, d, *J* = 5.1 Hz, -CH-O$\underline{H}$), 5.03 (1H, dd, *J* = 5.1, 2.8 Hz, -C$\underline{H}$-OH), 4.91 (1H, t, *J* = 5.6 Hz, -CH_2-O$\underline{H}$), 4.85 (2H, br s, H-6), 4.15 (2H, s, C_{13}-C$\underline{H}_2$-CONH), 4.09 (3H, s, C_9-OC$\underline{H}_3$), 4.07 (3H, s, C_{10}-OC$\underline{H}_3$), 4.06–4.01 (1H, m, -C$\underline{H}$-NH-CO-), 3.59 (1H, dt, *J* = 10.3, 6.8 Hz, -C$\underline{H}_2^a$-OH), 3.33 (1H, dt, *J* = 10.3, 5.2 Hz, -C$\underline{H}_2^b$-OH), 3.25 (2H, q, *J* = 6.8 Hz, β-C$\underline{H}_2$), 3.10 (2H, t, *J* =

5.9 Hz, H-5), 2.41–2.23 (2H, m, α-C$\underline{H}_2$); ^{13}C NMR (DMSO-d_6, 101 MHz) δ (ppm) 170.26 (-CH-NH-$\underline{C}$O-), 169.48 (-CH_2-NH-$\underline{C}$O-), 151.98 (NO_2-Ph_{para}), 150.34 (C-10), 149.38 (C-3), 146.71 (C-2), 146.28 (NO_2-Ph_{ipso}), 145.24 (C-8), 144.28 (C-9), 137.42 (C-13a), 134.01 (C-4a), 133.06 (C-12a), 127.96 (C-13), 127.50 (2C, NO_2-Ph_{meta}), 126.30 (C-11), 122.83 (2C, NO_2-Ph_{ortho}), 120.90 (C-8a), 120.80 (C-12), 120.17 (C-13b), 109.21 (C-1), 108.41 (C-4), 102.13 (O-$\underline{C}H_2$-O), 69.56 (-$\underline{C}$H-OH), 62.14 (C_9-O$\underline{C}H_3$), 60.55 (-$\underline{C}H_2$-OH), 57.06 (C_{10}-O$\underline{C}H_3$), 56.90 (C-6), 56.00 (-NH-$\underline{C}$H-), 37.45 (-NH-CO-$\underline{C}H_2$-C_{13}), 35.86 (β-CH_2), 35.07 (α-CH_2), 27.27 (C-5).

Detailed Synthesis of CAM-C3-BER

N-[(1R,2R)-1,3-dihydroxy-1-(4-nitrophenyl)propan-2-yl]-4-(2-[(13RS,13aRS)-9,10-dimethoxy-5,8,13,13a-tetrahydro-6H-benzo[g]-1,3-benzodioxolo[5,6-a]quinolizin-13-yl]acetamido)butanamide (CAM-C3-thBER)

CAM-C3-thBER was obtained as CAM-C8-thBER from 101 mg (0.232 mmol) of thBER-CH_2-COOH·HCl, 37 mg (0.323 mmol) of HOSu, 67 mg (0.323 mmol) of DCC, 88 μL (0.506 mmol) of DIPEA, and 104 mg (0.253 mmol) of CAM-C3-NH_2·TFA. The target product was isolated on a silica gel column eluting with a solvent system of $CHCl_3$:MeOH = 9:1. As a result, a light yellow solid was obtained. Yield: 55.4 mg (35%); TLC: R_f ($CHCl_3$:MeOH, 9:1) 0.31; LC-MS *m*/*z* calculated for $C_{35}H_{41}N_4O_{10}$ $(M+H)^+$: 677.28, found 677.53; t_R = 1.42 min; ^{1}H NMR (DMSO-d_6, 400 MHz) δ (ppm) 8.12 (2H, d, *J* = 8.5 Hz, *o*-H NO_2-Phe), 7.60–7.53 (3H, m, *m*-H NO_2-Phe, -CH_2-N$\underline{H}$-CO-), 7.42 (1H, d, *J* = 9.3 Hz, -CH-N$\underline{H}$-CO-), 6.90–6.73 (3H, m, H-12, H-11, H-1), 6.67 (1H, s, H-4), 5.95 (2H, s, O-C$\underline{H}_2$-O), 5.79 (1H, d, *J* = 4.8 Hz, -CH-O$\underline{H}$), 5.00 (1H, t, *J* = 3.8 Hz, -C$\underline{H}$-OH), 4.81 (1H, t, *J* = 5.6 Hz, -CH_2-O$\underline{H}$), 4.06 (1H, d, *J* = 15.7 Hz, H-8^a), 3.96 (1H, tdd, *J* = 8.5, 5.6, 2.7 Hz, -C$\underline{H}$-NH-CO-), 3.74 (3H, s, C_{10}-OC$\underline{H}_3$), 3.73 (3H, s, C_9-OC$\underline{H}_3$), 3.65–3.58 (2H, m, H-13, H-13a), 3.57–3.49 (1H, m, -C$\underline{H}_2{}^a$-OH), 3.41 (1H, d, *J* = 15.7 Hz, H-8^b), 3.27 (1H, dd, *J* = 10.8, 5.6 Hz, -C$\underline{H}_2{}^b$-OH), 3.07 (1H, br d, *J* = 8.4 Hz, H-6^a), 2.93–2.81 (1H, m, H-5^a), 2.78–2.70 (2H, m, γ-C$\underline{H}_2$), 2.56 (1H, d, *J* = 15.4 Hz, H-5^b), 2.43 (1H, t, *J* = 11.9 Hz, H-6^b), 2.22–2.13 (1H, m, α-C$\underline{H}_2{}^a$), 2.15–2.06 (1H, m, C_{13}-C$\underline{H}_2{}^a$-CO), 1.93–1.80 (2H, m, C_{13}-C$\underline{H}_2{}^b$-CO, α-C$\underline{H}_2{}^b$), 1.31 (2H, p, *J* = 7.3 Hz, β-C$\underline{H}_2$); ^{13}C NMR (DMSO-d_6, 101 MHz) δ (ppm) 171.31 (-CH-NH-$\underline{C}$O-), 170.28 (-CH_2-NH-$\underline{C}$O-), 151.93 (NO_2-Ph_{para}), 149.99 (C-10), 146.27 (NO_2-Ph_{ipso}), 145.89 (C-2), 145.42 (C-3), 144.30 (C-9), 132.29 (C-12a), 129.11 (C-4a), 128.67 (C-13b), 127.44 (C-8a), 127.28 (2C, NO_2-Ph_{meta}), 123.94 (C-12), 122.69 (2C, NO_2-Ph_{ortho}), 110.91 (C-11), 107.94 (C-4), 105.74 (C-1), 100.59 (O-$\underline{C}H_2$-O), 69.37 (-$\underline{C}$H-OH), 62.68 (C-13a), 60.52 (-$\underline{C}H_2$-OH), 59.40 (C_9-O$\underline{C}H_3$), 55.71 (-NH-$\underline{C}$H-), 55.62 (C_{10}-O$\underline{C}H_3$), 53.82 (C-8), 50.55 (C-6), 40.20–38.84 (2C, -NH-CO-$\underline{C}H_2$-C_{13}, γ-CH_2, overlapped with DMSO), 39.42 (C-13), 32.69 (α-CH_2), 28.96 (C-5), 25.37 (β-CH_2).

13-{[(3-{[(1R,2R)-1,3-dihydroxy-1-(4-nitrophenyl)propan-2-yl]carbamoyl}propyl)carbamoyl]methyl}-9,10-dimethoxy-5,6-dihydrobenzo[g]-1,3-benzodioxolo[5,6-a]quinolizinium hydroxide (CAM-C3-BER)

CAM-C3-BER was obtained as CAM-C8-BER from 40 mg (0.059 mmol) of CAM-C3-thBER and 21 mg (0.119 mmol) of NBS. The target product was isolated on a silica gel column eluting with a solvent system of $CHCl_3$:MeOH:NH_4OH = 65:25:4. As a result, a brown solid was obtained. Yield: 14 mg (34%); TLC: R_f ($CHCl_3$:MeOH:NH_4OH, 65:25:4) 0.51; LC-MS *m*/*z* calculated for $C_{35}H_{37}N_4O_{10}$ $(M)^+$: 673.25, found 673.35; t_R = 1.33 min; ^{1}H NMR (DMSO-d_6, 400

MHz) δ (ppm) 9.96 (1H, s, H-8), 8.66 (1H, t, *J* = 5.6 Hz, -CH_2-NH-CO-), 8.21 (1H, d, *J* = 9.3 Hz, H-11), 8.12 (2H, d, *J* = 8.6 Hz, *o*-H NO_2-Phe), 7.95 (1H, d, *J* = 9.3 Hz, H-12), 7.63 (1H, d, *J* = 9.2 Hz, -CH-NH-CO-), 7.60 (1H, s, H-1), 7.59 (2H, d, *J* = 8.6 Hz, *m*-H NO_2-Phe), 7.15 (1H, s, H-4), 6.15 (2H, s, O-CH_2-O), 5.89 (1H, d, *J* = 5.0 Hz, -CH-OH), 5.04 (1H, dd, *J* = 5.0, 2.5 Hz, -CH-OH), 4.91 (1H, t, *J* = 5.6 Hz, -CH_2-OH), 4.85 (2H, br s, H-6), 4.18 (2H, s, C_{13}-CH_2-CONH), 4.10 (3H, s, C_9-OCH_3), 4.07 (3H, s, C_{10}-OCH_3), 4.07–3.98 (1H, m, -CH-NH-CO-), 3.57 (1H, dt, *J* = 10.4, 7.1 Hz, -$CH_2{}^a$-OH), 3.32 (1H, dt, *J* = 10.4, 5.2 Hz, -$CH_2{}^b$-OH), 3.10 (2H, t, *J* = 6.0 Hz, H-5), 3.08–2.89 (2H, m, γ-CH_2), 2.11 (2H, t, *J* = 7.3 Hz, α-CH_2), 1.66–1.46 (2H, m, β-CH_2); ^{13}C NMR (DMSO-d_6, 101 MHz) δ (ppm) 171.67 (-CH-NH-CO-), 169.35 (-CH_2-NH-CO-), 152.09 (NO_2-Ph_{para}), 150.34 (C-10), 149.34 (C-3), 146.69 (C-2), 146.28 (NO_2-Ph_{ipso}), 145.17 (C-8), 144.23 (C-9), 137.41 (C-13a), 133.99 (C-4a), 133.16 (C-12a), 128.00 (C-13), 127.39 (2C, NO_2-Ph_{meta}), 126.27 (C-11), 122.81 (2C, NO_2-Ph_{ortho}), 120.94 (C-8a), 120.78 (C-12), 120.18 (C-13b), 109.24 (C-1), 108.37 (C-4), 102.11 (O-CH_2-O), 69.41 (-CH-OH), 62.07 (C_9-OCH_3), 60.62 (-CH_2-OH), 57.00 (C_{10}-OCH_3), 56.89 (C-6), 55.90 (-NH-CH-), 38.61 (γ-CH_2), 37.43 (-NH-CO-CH_2-C_{13}), 32.68 (α-CH_2), 27.26 (C-5), 25.34 (β-CH_2).

Detailed Synthesis of CAM-C5-BER

tert-butyl-N-(5-{[(1R,2R)-1,3-dihydroxy-1-(4-nitrophenyl)propan-2-yl]carbamoyl}pentyl)carbamate (CAM-C5-NH-Boc)

To a cold solution of 1000 mg (4.33 mmol) of *N*-Boc-6-aminocaproic acid and 648 mg (5.63 mmol) of HOSu in 9 mL of DMF, 1161 mg (5.63 mmol) of DCC was added at 0 °C. The mixture was stirred for 2 h at 0 °C and overnighted at RT. Then 1077 mg (4.33 mmol) of CAM·HCl in 6 mL of DMF and 981 μL (5.63 mmol) of DIPEA were added, and the resulted mixture was stirred at RT overnight. Then the reaction mixture was diluted with 200 μL of water. The mixture was then extracted with ethyl acetate (3×50 mL), and the combined organic extracts were washed with 0.2 M H_2SO_4 (3×25 mL), H_2O (1×25 mL), 5% $NaHCO_3$ (3×25 mL), saturated NaCl solution (1×25 mL). The organic layer was dried over anhydrous Na_2SO_4, and the volatiles were evaporated in vacuo. The target product was isolated on a silica gel column eluting with a solvent system of $CHCl_3$:MeOH = 9:1. The result was a yellowish oily substance. Yield: 942 mg (51%); TLC: R_f ($CHCl_3$:MeOH, 9:1) 0.41; LC-MS *m/z* calculated for $C_{20}H_{32}N_3O_7$ $(M+H)^+$: 426.22, found 426.49; t_R = 1.77 min; ^1H NMR ($CDCl_3$, 400 MHz) δ (ppm) 8.12 (2H, d, *J* = 7.8 Hz, *o*-H NO_2-Phe), 7.56 (2H, d, *J* = 8.4 Hz, *m*-H NO_2-Phe), 6.74 (1H, br s, -CH-NH-CO-), 5.19 (1H, d, *J* = 2.9 Hz, -CH-OH), 4.77 (1H, br s, -CH_2-NH-Boc), 4.19 (1H, dq, *J* = 8.9, 4.9 Hz, -CH-NH-CO-), 3.80 (1H, dd, *J* = 11.3, 5.8 Hz, -$CH_2{}^a$-OH), 3.72 (1H, dd, *J* = 11.3, 4.1 Hz, -$CH_2{}^b$-OH), 2.97 (2H, t, *J* = 7.0 Hz, ε-CH_2), 2.08 (2H, t, *J* = 7.3 Hz, α-CH_2), 1.47–1.30 (4H, m, β-, δ-CH_2), 1.39 (9H, s, -CH_3), 1.18–1.05 (2H, m, γ-CH_2); ^{13}C NMR ($CDCl_3$, 101 MHz) δ (ppm) 174.61 (-CH-NH-CO-), 156.50 (-CH_2-NH-CO-), 149.51 (NO_2-Ph_{para}), 147.25 (NO_2-Ph_{ipso}), 127.03 (2C, NO_2-Ph_{meta}), 123.46 (2C, NO_2-Ph_{ortho}), 79.67 (-C$(CH_3)_3$), 71.94 (-CH-OH), 62.72 (-CH_2-OH), 56.17 (-NH-CH-), 40.43 (ε-CH_2), 36.17 (α-CH_2), 29.55 (δ-CH_2), 28.50 (3C, -CH_3), 25.98 (γ-CH_2), 25.17 (β-CH_2).

6-amino-N-[(1R,2R)-1,3-dihydroxy-1-(4-nitrophenyl)propan-2-yl]hexanamide (CAM-C5-NH_2)

840 mg (1.97 mmol) of CAM-C5-NH-Boc was dissolved in 10 mL of 50% TFA in CH_2Cl_2 at RT, the obtained mixture was stirred for 30 min. Then the volatiles were evaporated in vacuo,

the residue was precipitated with diethyl ether, the ether was then decanted, and the precipitate was washed with diethyl ether. The target product was isolated on a silica gel column eluting with a solvent system of $CHCl_3$:MeOH:NH_4OH = 65:25:4. The result was a light yellow oily substance. Yield: 640 mg (100%); TLC: R_f ($CHCl_3$:MeOH:NH_4OH, 65:25:4) 0.38; LC-MS *m/z* calculated for $C_{15}H_{24}N_3O_5$ $(M+H)^+$: 326.17, found 326.34; t_R = 0.54 min; ^{1}H NMR (DMSO-d_6, 400 MHz) δ (ppm) 8.15 (2H, d, *J* = 8.4 Hz, *o*-H NO_2-Phe), 7.81 (2H, br s, -N$\underline{H}_2$), 7.57 (2H, d, *J* = 8.4 Hz, *m*-H NO_2-Phe), 7.52 (1H, d, *J* = 9.2 Hz, -CH-N$\underline{H}$-CO-), 5.88 (1H, d, *J* = 4.9 Hz, -CH-O$\underline{H}$), 5.01 (1H, d, *J* = 3.7 Hz, -C$\underline{H}$-OH), 4.81 (1H, s, -CH_2-O$\underline{H}$), 4.00 (1H, ddt, *J* = 10.4, 9.1, 4.5 Hz, -C$\underline{H}$-NH-CO-), 3.55 (1H, t, *J* = 9.2 Hz, -C$\underline{H}_2^a$-OH), 3.29 (1H, dd, *J* = 10.4, 5.8 Hz, -C$\underline{H}_2^b$-OH), 2.68 (2H, t, *J* = 7.6 Hz, ε-C$\underline{H}_2$), 1.97 (2H, t, *J* = 7.4 Hz, α-C$\underline{H}_2$), 1.42 (2H, p, *J* = 7.6 Hz, δ-C$\underline{H}_2$), 1.28 (2H, p, *J* = 7.5 Hz, β-C$\underline{H}_2$), 1.13–0.97 (2H, m, γ-C$\underline{H}_2$); ^{13}C NMR (DMSO-d_6, 101 MHz) δ (ppm) 171.98 (-CH-NH-$\underline{C}$O-), 152.25 (NO_2-Ph_{para}), 146.32 (NO_2-Ph_{ipso}), 127.43 (2C, NO_2-Ph_{meta}), 122.80 (2C, NO_2-Ph_{ortho}), 69.44 (-$\underline{C}$H-OH), 60.64 (-$\underline{C}H_2$-OH), 55.82 (-NH-CH-), 38.66 (ε-CH_2), 34.84 (α-CH_2), 26.79 (δ-CH_2), 25.30 (γ-CH_2), 24.80 (β-CH_2).

N-[(1R,2R)-1,3-dihydroxy-1-(4-nitrophenyl)propan-2-yl]-6-(2-[(13RS,13aRS)-9,10-dimethoxy-5,8,13,13a-tetrahydro-6H-benzo[g]-1,3-benzodioxolo[5,6-a]quinolizin-13-yl]acetamido)hexanamide (CAM-C5-thBER)

CAM-C5-thBER was obtained as CAM-C8-thBER from 225 mg (0.518 mmol) of thBER-CH_2-COOH·HCl, 85 mg (0.74 mmol) of HOSu, 152 mg (0.74 mmol) of DCC, 197 μL (1.13 mmol) of DIPEA, and 185 mg (0.57 mmol) of CAM-C5-NH_2. The target product was isolated on a silica gel column eluting with a solvent system of $CHCl_3$:MeOH = 9:1. As a result, a light yellow solid was obtained. Yield: 168 mg (46%); TLC: R_f ($CHCl_3$:MeOH, 9:1) 0.37; LC-MS *m/z* calculated for $C_{37}H_{45}N_4O_{10}$ $(M+H)^+$: 705.31, found 705.29; t_R = 1.50 min; ^{1}H NMR (DMSO-d_6, 400 MHz) δ (ppm) 8.16 & 8.15 (2H, d, *J* = 8.7 Hz, *o*-H NO_2-Phe), 7.570 & 7.567 (2H, d, *J* = 8.7 Hz, *m*-H NO_2-Phe), 7.50 (1H, t, *J* = 5.7 Hz, -CH_2-N$\underline{H}$-CO-), 7.44 (1H, d, *J* = 9.2 Hz, -CH-N$\underline{H}$-CO-), 6.85 & 6.84 (1H, d, *J* = 8.6 Hz, H-12), 6.83 (1H, s, H-1), 6.80 (1H, d, *J* = 8.6 Hz, H-11), 6.67 (1H, s, H-4), 5.95 (2H, s, O-C$\underline{H}_2$-O), 5.80 (1H, d, *J* = 4.5 Hz, -CH-O$\underline{H}$), 5.02 (1H, dd, *J* = 4.5, 2.4 Hz, -C$\underline{H}$-OH), 4.83 (1H, dd, *J* = 6.4, 4.7 Hz, -CH_2-O$\underline{H}$), 4.06 (1H, d, *J* = 16.0 Hz, H-8^a), 3.99 (1H, dddd, *J* = 9.2, 8.4, 5.4, 2.4 Hz, -C$\underline{H}$-NH-CO-), 3.74 (3H, s, C_{10}-OC$\underline{H}_3$), 3.72 (3H, s, C_9-OC$\underline{H}_3$), 3.65–3.58 (2H, m, H-13, H-13a), 3.57–3.50 (1H, m, -C$\underline{H}_2^a$-OH), 3.40 (1H, d, *J* = 16.0 Hz, H-8^b), 3.28 (1H, dt, *J* = 10.5, 5.4 Hz, -C$\underline{H}_2^b$-OH), 3.06 (1H, dd, *J* = 11.0, 4.4 Hz, H-6^a), 2.93–2.70 (3H, m, ε-C$\underline{H}_2$, H-5^a), 2.56 (1H, d, *J* = 15.5 Hz, H-5^b), 2.42 (1H, ddd, *J* = 13.9, 11.0, 2.9 Hz, H-6^b), 2.11 (1H, dd, *J* = 14.4, 9.6 Hz, C_{13}-C$\underline{H}_2^a$-CO), 1.93 (2H, td, *J* = 7.2, 2.3 Hz, α-C$\underline{H}_2$), 1.80 (1H, dd, *J* = 14.4, 2.6 Hz, C_{13}-C$\underline{H}_2^b$-CO), 1.28–1.06 (4H, m, β-C$\underline{H}_2$, δ-C$\underline{H}_2$), 0.95–0.80 (2H, m, γ-C$\underline{H}_2$); ^{13}C NMR (DMSO-d_6, 101 MHz) δ (ppm) 172.03 (-CH-NH-$\underline{C}$O-), 171.24 (-CH_2-NH-$\underline{C}$O-), 152.14 (NO_2-Ph_{para}), 150.05 (C-10), 146.26 (NO_2-Ph_{ipso}), 145.93 (C-2), 145.44 (C-3), 144.28 (C-9), 132.32 (C-12a), 129.16 (C-4a), 128.75 (C-13b), 127.52 (C-8a), 127.34 (2C, NO_2-Ph_{meta}), 124.04 (C-12), 122.77 (2C, NO_2-Ph_{ortho}), 110.82 (C-11), 108.03 (C-4), 105.81 (C-1), 100.62 (O-$\underline{C}H_2$-O), 69.33 (-$\underline{C}$H-OH), 62.73 (C-13a), 60.56 (-$\underline{C}H_2$-OH), 59.44 (C_9-O$\underline{C}H_3$), 55.72 (-NH-$\underline{C}$H-), 55.62 (C_{10}-O$\underline{C}H_3$), 53.89 (C-8), 50.58 (C-6), 39.48 (C-13), 38.89 (-NH-CO-$\underline{C}H_2$-C_{13}), 38.26 (ε-CH_2), 35.02 (α-CH_2), 29.13 (C-5), 28.77 (δ-CH_2), 25.82 (γ-CH_2), 25.00 (β-CH_2).

13-{[(5-{[(1R,2R)-1,3-dihydroxy-1-(4-nitrophenyl)propan-2-yl]carbamoyl}pentyl)carbamoyl]methyl}-9,10-dimethoxy-5,6-dihydrobenzo[g]-1,3-benzodioxolo[5,6-a]quinolizinium hydroxide (CAM-C5-BER)

CAM-C5-BER was obtained as CAM-C8-BER from 115 mg (0.163 mmol) of CAM-C5-thBER and 58 mg (0.326 mmol) of NBS. The target product was isolated on a silica gel column eluting with a solvent system of $CHCl_3$:MeOH:NH_4OH = 65:25:4. As a result, a mustard-colored solid was obtained. Yield: 66 mg (56%); TLC: R_f ($CHCl_3$:MeOH:NH_4OH, 65:25:4) 0.44; LC-MS *m/z* calculated for $C_{37}H_{41}N_4O_{10}$ $(M)^+$: 701.28, found 701.43; t_R = 1.43 min; ^{1}H NMR (DMSO-d_6, 400 MHz) δ (ppm) 9.97 (1H, s, H-8), 8.76 (1H, t, *J* = 5.5 Hz, -CH_2-N$\underline{H}$-CO-), 8.22 (1H, d, *J* = 9.3 Hz, H-11), 8.14 (2H, d, *J* = 8.6 Hz, *o*-H NO_2-Phe), 7.95 (1H, d, *J* = 9.3 Hz, H-12), 7.61 (1H, s, H-1), 7.59 (2H, d, *J* = 8.6 Hz, *m*-H NO_2-Phe), 7.57 (1H, d, *J* = 9.4 Hz, -CH-N$\underline{H}$-CO-), 7.16 (1H, s, H-4), 6.15 (2H, br s, O-C$\underline{H}_2$-O), 5.86 (1H, d, *J* = 5.3 Hz, -CH-O$\underline{H}$), 5.03 (1H, dd, *J* = 5.3, 2.5 Hz, -C$\underline{H}$-OH), 4.90 (1H, t, *J* = 5.6 Hz, -CH_2-O$\underline{H}$), 4.85 (2H, br s, H-6), 4.20 (2H, s, C_{13}-C$\underline{H}_2$-CONH), 4.10 (3H, s, C_9-OC$\underline{H}_3$), 4.06 (3H, s, C_{10}-OC$\underline{H}_3$), 4.05–3.96 (1H, m, -C$\underline{H}$-NH-CO-), 3.56 (1H, td, *J* = 10.4, 6.5 Hz, -C$\underline{H}_2^a$-OH), 3.29 (1H, dt, *J* = 10.4, 5.3 Hz, -C$\underline{H}_2^b$-OH), 3.18–3.02 (4H, m, H-5, ε-C$\underline{H}_2$), 2.01 (2H, t, *J* = 7.4 Hz, α-C$\underline{H}_2$), 1.40 (2H, p, *J* = 7.0 Hz, δ-C$\underline{H}_2$), 1.37–1.26 (2H, m, β-C$\underline{H}_2$), 1.17–1.05 (2H, m, γ-C$\underline{H}_2$); ^{13}C NMR (DMSO-d_6, 101 MHz) δ (ppm) 172.10 (-CH-NH-$\underline{C}$O-), 169.33 (-CH_2-NH-$\underline{C}$O-), 152.19 (NO_2-Ph_{para}), 150.36 (C-10), 149.33 (C-3), 146.69 (C-2), 146.22 (NO_2-Ph_{ipso}), 145.18 (C-8), 144.23 (C-9), 137.39 (C-13a), 133.99 (C-4a), 133.14 (C-12a), 128.04 (C-13), 127.40 (2C, NO_2-Ph_{meta}), 126.24 (C-11), 122.76 (2C, NO_2-Ph_{ortho}), 120.94 (C-8a), 120.82 (C-12), 120.18 (C-13b), 109.22 (C-1), 108.38 (C-4), 102.10 (O-$\underline{C}H_2$-O), 69.41 (-$\underline{C}$H-OH), 62.11 (C_9-O$\underline{C}H_3$), 60.56 (-$\underline{C}H_2$-OH), 57.07 (C_{10}-O$\underline{C}H_3$), 56.88 (C-6), 55.77 (-NH-$\underline{C}$H-), 38.89 (ε-CH_2), 37.46 (-NH-CO-$\underline{C}H_2$-C_{13}), 35.09 (α-CH_2), 28.77 (δ-CH_2), 27.26 (C-5), 26.00 (γ-CH_2), 25.08 (β-CH_2).

Detailed Synthesis of CAM-C8-NH_2

9-[(tert-butoxycarbonyl)amino]nonanoic acid (Boc-NH-(CH_2)$_8$-COOH)

2 g (11.5 mmol) of 9-aminopelargonic acid, 3 mL (17.3 mmol) of DIPEA, and 3.96 mL (17.3 mmol) of Boc_2O were dissolved in a mixture of 10 mL of anhydrous CH_2Cl_2 and 20 mL of DMF, the obtained mixture was stirred for 3 h at RT. CH_2Cl_2 was then evaporated in vacuo, the mixture was diluted with 200 μL of water and washed with petroleum ether (2×50 mL). 1N aqueous H_2SO_4 was added dropwise to pH 6. The mixture was then extracted with ethyl acetate (3×50 mL), and the combined organic extracts were washed with 1N H_2SO_4 (3×50 mL) and saturated NaCl solution (1×50 mL). The organic layer was dried over anhydrous Na_2SO_4, and the volatiles were evaporated in vacuo. As a result, a white solid was obtained. Yield: 1.62 mg (52%); TLC: R_f ($CHCl_3$:MeOH, 9:1) 0.62; LC-MS *m/z* calculated for $C_{14}H_{27}NNaO_4$ $(M+Na)^+$: 296.18, found 296.33; t_R = 2.27 min; ^{1}H NMR (DMSO-d_6, 400 MHz) δ (ppm) 11.90 (1H, s, -COO$\underline{H}$), 6.68 (1H, t, *J* = 5.7 Hz, -N$\underline{H}$-Boc), 2.89 (2H, q, *J* = 6.8 Hz, θ-C$\underline{H}_2$), 2.18 (2H, t, *J* = 7.3 Hz, α-C$\underline{H}_2$), 1.48 (2H, p, *J* = 7.3 Hz, η-C$\underline{H}_2$), 1.37 (9H, s, -C$\underline{H}_3$), 1.37–1.31 (2H, m, β-C$\underline{H}_2$), 1.29–1.18 (8H, m, γ-, δ-, ε-, ζ-C$\underline{H}_2$); ^{13}C NMR (DMSO-d_6, 101 MHz) δ (ppm) 174.39 (-$\underline{C}$OOH), 155.54 (-NH-$\underline{C}$O-), 77.21 (-$\underline{C}(CH_3)_3$), 39.79 (θ-CH_2), 33.64 (α-CH_2), 29.41 (η-CH_2), 28.66 (δ-CH_2), 28.55 (ε-CH_2), 28.46 (γ-CH_2), 28.23 (3C, -CH_3), 26.18 (ζ-CH_2), 24.45 (β-CH_2).

tert-butyl-N-(8-{[(1R,2R)-1,3-dihydroxy-1-(4-nitrophenyl)propan-2-yl]carbamoyl}octyl)carbamate (CAM-C8-NH-Boc)

CAM-C8-NH-Boc was obtained as CAM-C5-NH-Boc from 1000 mg (3.663 mmol) of Boc-NH-$(CH_2)_8$-COOH, 548 mg (4.763 mmol) of HOSu, 982 mg (4.76 mmol) of DCC, 911 mg (3.66 mmol) of CAM·HCl, and 638 μL (3.663 mmol) of DIPEA. The target product was isolated on a silica gel column eluting with a solvent system of $CHCl_3$:MeOH = 9:1. As a result, a white solid was obtained. Yield: 1.488 mg (87%); TLC: R_f ($CHCl_3$:MeOH, 9:1) 0.47; LC-MS *m*/*z* calculated for $C_{23}H_{38}N_3O_7$ $(M+H)^+$: 468.27, found 468.54; t_R = 2.18 min; ^{1}H NMR ($CDCl_3$, 400 MHz) δ (ppm) 8.20–8.12 (2H, m, *o*-H NO_2-Phe), 7.56 (2H, d, *J* = 8.7 Hz, *m*-H NO_2-Phe), 6.44 (1H, d, *J* = 8.8 Hz, -CH-NH-CO-), 5.20 (1H, t, *J* = 3.7 Hz, -CH-OH), 5.01 (1H, br s, -NH-Boc), 4.70 (1H, br s, -CH-OH), 4.19 (1H, dt, *J* = 8.7, 4.3 Hz, -CH-NH-CO-), 4.04 (1H, br s, -CH_2-OH), 3.83–3.75 (2H, m, -CH_2-OH), 3.05 (2H, q, *J* = 6.8 Hz, θ-CH_2), 2.14–2.01 (2H, m, α-CH_2), 1.44–1.38 (11H, m, η-CH_2, -CH_3), 1.31–1.13 (8H, m, β-, δ-, ε-, ζ-CH_2), 1.13–1.03 (2H, m, γ-CH_2); ^{13}C NMR ($CDCl_3$, 101 MHz) δ (ppm) 174.45 (-CH-NH-CO-), 156.48 (-CH_2-NH-CO-), 149.47 (NO_2-Ph_{para}), 147.29 (NO_2-Ph_{ipso}), 126.91 (2C, NO_2-Ph_{meta}), 123.48 (2C, NO_2-Ph_{ortho}), 79.43 (-$C(CH_3)_3$), 72.43 (-CH-OH), 63.19 (-CH_2-OH), 55.96 (-NH-CH-), 40.63 (θ-CH_2), 36.54 (α-CH_2), 29.96 (η-CH_2), 29.05 (δ-CH_2), 28.92 (ε-CH_2), 28.78 (γ-CH_2), 28.52 (3C, -CH_3), 26.61 (ζ-CH_2), 25.59 (β-CH_2).

9-amino-N-[(1R,2R)-1,3-dihydroxy-1-(4-nitrophenyl)propan-2-yl]nonanamide (CAM-C8-NH_2)

CAM-C8-NH_2 was obtained as CAM-C5-NH_2 from 1388 mg (2.97 mmol) of CAM-C8-NH-Boc and 10 mL of 50% TFA in CH_2Cl_2. The target product was isolated on a silica gel column eluting with a solvent system of $CHCl_3$:MeOH:NH_4OH = 65:25:4. The result was a beige oily substance. Yield: 900 mg (83%); TLC: R_f ($CHCl_3$:MeOH:NH_4OH, 65:25:4) 0.32; LC-MS *m*/*z* calculated for $C_{18}H_{30}N_3O_5$ $(M+H)^+$: 368.22, found 368.01; t_R = 0.92 min; ^{1}H NMR (DMSO-d_6, 400 MHz) δ (ppm) 8.14 (2H, d, *J* = 8.7 Hz, *o*-H NO_2-Phe), 7.58 (2H, d, *J* = 8.7 Hz, *m*-H NO_2-Phe), 7.47 (1H, d, *J* = 9.2 Hz, -CH-NH-CO-), 5.04 (1H, d, *J* = 2.5 Hz, -CH-OH), 4.78 (4H, br s, -NH_2, -CH-OH, -CH_2-OH), 4.01 (1H, tdd, *J* = 8.5, 5.6, 2.5 Hz, -CH-NH-CO-), 3.55 (1H, dd, *J* = 10.3, 8.3 Hz, -CH_2^a-OH), 3.30 (1H, dd, *J* = 10.3, 5.7 Hz, -CH_2^b-OH), 2.64 (2H, t, *J* = 7.3 Hz, θ-CH_2), 2.06–1.87 (2H, m, α-CH_2), 1.41 (2H, p, *J* = 7.4 Hz, η-CH_2), 1.33–1.16 (4H, m, β-CH_2, ζ-CH_2), 1.15–1.04 (4H, m, δ-CH_2, ε-CH_2), 1.00–0.83 (2H, m, γ-CH_2); ^{13}C NMR (DMSO-d_6, 101 MHz) δ (ppm) 172.09 (-CH-NH-CO-), 152.26 (NO_2-Ph_{para}), 146.21 (NO_2-Ph_{ipso}), 127.34 (2C, NO_2-Ph_{meta}), 122.73 (2C, NO_2-Ph_{ortho}), 69.30 (-CH-OH), 60.60 (-CH_2-OH), 55.71 (-NH-CH-), 39.98 (θ-CH_2), 35.07 (α-CH_2), 29.62 (η-CH_2), 28.79 (δ-CH_2), 28.61 (ε-CH_2), 28.30 (γ-CH_2), 26.12 (ζ-CH_2), 25.30 (β-CH_2).

Detailed Synthesis of CH_3-C5-BER

The scheme for the synthesis of CH_3-Cn-BER is represented in Figure S1.

2-[(13RS,13aRS)-9,10-dimethoxy-5,8,13,13a-tetrahydro-6H-benzo[g]-1,3-benzodioxolo[5,6-a]quinolizin-13-yl]-N-hexylacetamide (CH_3-C5-thBER)

To a cold solution of 200 mg (0.461 mmol) of thBER-CH_2-COOH·HCl in 20 mL of DMF, 124 mg (0.6 mmol) of DCC, 67 μL (0.51 mmol) of hexylamine, and 241 μL (1.383 mmol) of DIPEA were added at 0 °C. The mixture was stirred for 3 h at 0 °C and overnighted at 4 °C. Then the reaction mixture was diluted with 200 μL of water, extracted with ethyl acetate (3×50

mL), and the combined organic extracts were washed with saturated NaCl solution (1×50 mL). The organic layer was dried over anhydrous Na_2SO_4, and the volatiles were evaporated in vacuo. The target product was isolated on a silica gel column eluting with a solvent system of $CHCl_3$:MeOH = 40:1. As a result, a light yellow solid was obtained. Yield: 115 mg (52%); TLC: R_f ($CHCl_3$:MeOH, 40:1) 0.37; LC-MS *m/z* calculated for $C_{28}H_{37}N_2O_5$ $(M+H)^+$: 481.27, found 481.10; t_R = 1.98 min; ^1H NMR (DMSO-d_6, COSY, ROESY, 600 MHz) δ (ppm) 7.52 (1H, t, *J* = 5.6 Hz, -N<u>H</u>-), 6.86 (1H, d, *J* = 8.5 Hz, H-12), 6.82 (1H, s, H-1), 6.80 (1H, d, *J* = 8.5 Hz, H-11), 6.67 (1H, s, H-4), 5.95 (2H, d, *J* = 1.3 Hz, O-C<u>H</u>$_2$-O), 4.06 (1H, d, *J* = 15.9 Hz, H-8^a), 3.75 (3H, s, C_{10}-OC<u>H</u>$_3$), 3.72 (3H, s, C_9-OC<u>H</u>$_3$), 3.62 (1H, dt, *J* = 10.0, 2.9 Hz, H-13), 3.59 (1H, d, *J* = 3.4 Hz, H-13a), 3.40 (1H, d, *J* = 15.9 Hz, H-8^b), 3.06 (1H, ddd, *J* = 10.9, 5.0, 1.9 Hz, H-6^a), 2.96 (1H, dq, *J* = 13.1, 6.6 Hz, 1′-CH_2^a), 2.91–2.85 (1H, m, H-5^a), 2.86–2.80 (1H, m, 1′-CH_2^b), 2.56 (1H, d, *J* = 15.9 Hz, H-5^b), 2.42 (1H, td, *J* = 12.0, 2.9 Hz, H-6^b), 2.13 (1H, dd, *J* = 14.5, 10.0 Hz, C_{13}-C<u>H</u>$_2^a$-CO), 1.81 (1H, dd, *J* = 14.5, 3.0 Hz, C_{13}-C<u>H</u>$_2^b$-CO), 1.29–1.23 (2H, m, 2′-CH_2), 1.23–1.20 (2H, m, 5′-CH_2), 1.20–1.14 (2H, m, 4′-CH_2), 1.14–1.07 (2H, m, 3′-CH_2), 0.83 (3H, t, *J* = 7.1 Hz, 6′-CH_3); ^{13}C NMR (DMSO-d_6, HMBC, HSQC, 151 MHz) δ (ppm) 171.76 (-CH_2-NH-<u>C</u>O-), 150.57 (C-10), 146.51 (C-2), 145.85 (C-3), 144.82 (C-9), 132.84 (C-12a), 129.65 (C-4a), 129.28 (C-13b), 128.08 (C-8a), 124.67 (C-12), 111.37 (C-11), 108.66 (C-4), 106.43 (C-1), 101.26 (O-<u>C</u>H$_2$-O), 63.40 (C-13a), 60.02 (C_9-O<u>C</u>H$_3$), 56.19 (C_{10}-O<u>C</u>H$_3$), 54.53 (C-8), 51.21 (C-6), 40.11 (C-13), 39.54 (-NH-CO-<u>C</u>H$_2$-C_{13}), 39.10 (1′-CH_2), 31.65 (4′-CH_2), 29.73 (C-5), 29.62 (2′-CH_2), 26.72 (3′-CH_2), 22.68 (5′-CH_2), 14.53 (6′-CH_3).

13-[(hexylcarbamoyl)methyl]-9,10-dimethoxy-5,6-dihydrobenzo[g]-1,3-benzodioxolo[5,6-a]quinolizinium hydroxide (CH_3-C5-BER)

CH_3-C5-BER was obtained as CAM-C8-BER from 71.5 mg (0.149 mmol) of CH_3-C5-thBER and 53 mg (0.298 mmol) of NBS. The target product was isolated on a silica gel column eluting with a solvent system of $CHCl_3$:MeOH:NH_4OH = 6:1:0.1. As a result, a bright yellow solid was obtained. Yield: 56 mg (76%); TLC: R_f ($CHCl_3$:MeOH:NH_4OH, 6:1:0.1) 0.35; LC-MS *m/z* calculated for $C_{28}H_{33}N_2O_5$ $(M)^+$: 477.24, found 477.24; t_R = 1.87 min; ^1H NMR (DMSO-d_6, 400 MHz) δ (ppm) 9.98 (1H, s, H-8), 8.82 (1H, t, *J* = 5.6 Hz, -CH_2-N<u>H</u>-CO-), 8.21 (1H, d, *J* = 9.4 Hz, H-11), 7.98 (1H, d, *J* = 9.4 Hz, H-12), 7.62 (1H, s, H-1), 7.17 (1H, s, H-4), 6.16 (2H, s, O-C<u>H</u>$_2$-O), 4.86 (2H, br s, H-6), 4.22 (2H, s, C_{13}-C<u>H</u>$_2$-CONH), 4.10 (3H, s, C_9-OC<u>H</u>$_3$), 4.07 (3H, s, C_{10}-OC<u>H</u>$_3$), 3.19 (2H, q, *J* = 6.7 Hz, 1′-C<u>H</u>$_2$), 3.10 (2H, t, *J* = 5.9 Hz, H-5), 1.50 (2H, p, *J* = 7.1 Hz, 2′-C<u>H</u>$_2$), 1.39–1.20 (6H, m, 3′-, 4′-, 5′-C<u>H</u>$_2$), 0.87 (3H, t, *J* = 6.9 Hz, 6′-C<u>H</u>$_2$); ^{13}C NMR (DMSO-d_6, 101 MHz) δ (ppm) 169.34 (-CH_2-NH-<u>C</u>O-), 150.32 (C-10), 149.31 (C-3), 146.67 (C-2), 145.22 (C-8), 144.25 (C-9), 137.39 (C-13a), 134.02 (C-4a), 133.12 (C-12a), 128.03 (C-13), 126.13 (C-11), 120.93 (C-8a), 120.83 (C-12), 120.17 (C-13b), 109.18 (C-1), 108.39 (C-4), 102.09 (O-<u>C</u>H$_2$-O), 62.11 (C_9-O<u>C</u>H$_3$), 57.05 (C_{10}-O<u>C</u>H$_3$), 56.87 (C-6), 39.00 (1′-CH_2), 37.46 (-NH-CO-<u>C</u>H$_2$-C_{13}), 31.03 (4′-CH_2), 29.02 (2′-CH_2), 27.23 (C-5), 26.18 (3′-CH_2), 22.12 (5′-CH_2), 13.98 (6′-CH_3).

Detailed Synthesis of CH_3-C8-BER

2-[(13RS,13aRS)-9,10-dimethoxy-5,8,13,13a-tetrahydro-6H-benzo[g]-1,3-benzodioxolo[5,6-a]quinolizin-13-yl]-N-nonylacetamide (CH_3-C8-thBER)

CH$_3$-C8-thBER was obtained as CH$_3$-C5-thBER from 165 mg (0.380 mmol) of thBER-CH$_2$-COOH·HCl, 102 mg (0.49 mmol) of DCC, 85 mg (0.38 mmol) of nonylammonium bromide, and 198 µL (1.140 mmol) of DIPEA. The target product was isolated on a silica gel column eluting with a solvent system of CHCl$_3$:MeOH = 40:1. As a result, a light yellow solid was obtained. Yield: 146 mg (74%); TLC: R_f (CHCl$_3$:MeOH, 40:1) 0.34; LC-MS *m/z* calculated for $C_{31}H_{43}N_2O_5$ $(M+H)^+$: 523.32, found 523.16; t_R = 2.40 min; ^{1}H NMR (DMSO-d_6, COSY, ROESY, 600 MHz) δ (ppm) 7.51 (1H, t, *J* = 5.6 Hz, -NH-), 6.85 (1H, d, *J* = 8.5 Hz, H-12), 6.83 (1H, s, H-1), 6.80 (1H, d, *J* = 8.5 Hz, H-11), 6.68 (1H, s, H-4), 5.96 (1H, d, *J* = 1.1 Hz, O-CH$_2^a$-O), 5.95 (1H, d, *J* = 1.1 Hz, O-CH$_2^b$-O), 4.06 (1H, d, *J* = 15.9 Hz, H-8^a), 3.75 (3H, s, C$_{10}$-OCH$_3$), 3.72 (3H, s, C$_9$-OCH$_3$), 3.62 (1H, dt, *J* = 9.5, 2.7 Hz, H-13), 3.61 (1H, d, *J* = 4.4 Hz, H-13a), 3.41 (1H, d, *J* = 15.9 Hz, H-8^b), 3.06 (1H, ddd, *J* = 10.2, 5.0, 1.8 Hz, H-6^a), 2.95 (1H, dq, *J* = 13.0, 6.6 Hz, 1′-CH$_2^a$), 2.90–2.84 (1H, m, H-5^a), 2.86–2.79 (1H, m, 1′-CH$_2^b$), 2.57 (1H, d, *J* = 15.5 Hz, H-5^b), 2.42 (1H, td, *J* = 11.5, 2.9 Hz, H-6^b), 2.12 (1H, dd, *J* = 14.4, 9.8 Hz, C$_{13}$-CH$_2^a$-CO), 1.80 (1H, dd, *J* = 14.4, 2.9 Hz, C$_{13}$-CH$_2^b$-CO), 1.28–1.16 (12H, m, 2′-, 4′-, 5′-, 6′-, 7′-, 8′-CH$_2$), 1.13–1.07 (2H, m, 3′-CH$_2$), 0.84 (3H, t, *J* = 7.0 Hz, 9′-CH$_3$); ^{13}C NMR (DMSO-d_6, HMBC, HSQC, 151 MHz) δ (ppm) 171.72 (-CH$_2$-NH-CO-), 150.61 (C-10), 146.23 (C-2), 145.94 (C-3), 144.78 (C-9), 132.84 (C-12a), 129.67 (C-4a), 129.28 (C-13b), 128.11 (C-8a), 124.60 (C-12), 111.30 (C-11), 108.60 (C-4), 106.35 (C-1), 101.20 (O-CH$_2$-O), 63.25 (C-13a), 60.04 (C$_9$-OCH$_3$), 56.21 (C$_{10}$-OCH$_3$), 54.47 (C-8), 51.13 (C-6), 40.06 (C-13), 39.45 (-NH-CO-CH$_2$-C$_{13}$), 39.02 (1′-CH$_2$), 31.88 (7′-CH$_2$), 29.67 (C-5), 29.55 (2′-CH$_2$), 29.50, 29.44, 29.25 (4′-, 5′-, 6′-CH$_2$), 26.98 (3′-CH$_2$), 22.68 (8′-CH$_2$), 14.51 (9′-CH$_3$).

13-[(nonylcarbamoyl)methyl]-9,10-dimethoxy-5,6-dihydrobenzo[g]-1,3-benzodioxolo[5,6-a]quinolizinium hydroxide (CH$_3$-C8-BER)

CH$_3$-C8-BER was obtained as CAM-C8-BER from 106 mg (0.203 mmol) of CH$_3$-C8-thBER and 72 mg (0.406 mmol) of NBS. The target product was isolated on a silica gel column eluting with a solvent system of CHCl$_3$:MeOH:NH$_4$OH = 6:1:0.1. As a result, a bright yellow solid was obtained. Yield: 73 mg (67%); TLC: R_f (CHCl$_3$:MeOH:NH$_4$OH, 6:1:0.1) 0.31; LC-MS *m/z* calculated for $C_{31}H_{39}N_2O_5$ $(M)^+$: 519.28, found 519.17; t_R = 2.32 min; ^{1}H NMR (DMSO-d_6, 400 MHz) δ (ppm) 9.98 (1H, s, H-8), 8.78 (1H, t, *J* = 5.6 Hz, -CH$_2$-NH-CO-), 8.21 (1H, d, *J* = 9.4 Hz, H-11), 7.98 (1H, d, *J* = 9.4 Hz, H-12), 7.63 (1H, s, H-1), 7.17 (1H, s, H-4), 6.16 (2H, s, O-CH$_2$-O), 4.85 (2H, br s, H-6), 4.22 (2H, s, C$_{13}$-CH$_2$-CONH), 4.10 (3H, s, C$_9$-OCH$_3$), 4.08 (3H, s, C$_{10}$-OCH$_3$), 3.18 (2H, q, *J* = 6.6 Hz, 1′-CH$_2$), 3.10 (2H, t, *J* = 5.9 Hz, H-5), 1.50 (2H, p, *J* = 7.0 Hz, 2′-CH$_2$), 1.34–1.19 (12H, m, 3′-, 4′-, 5′-, 6′-, 7′-, 8′-CH$_2$), 0.85 (3H, t, *J* = 6.9 Hz, 9′-CH$_2$); ^{13}C NMR (DMSO-d_6, 101 MHz) δ (ppm) 169.33 (-CH$_2$-NH-CO-), 150.32 (C-10), 149.31 (C-3), 146.67 (C-2), 145.22 (C-8), 144.26 (C-9), 137.40 (C-13a), 134.02 (C-4a), 133.12 (C-12a), 128.04 (C-13), 126.13 (C-11), 120.94 (C-8a), 120.82 (C-12), 120.17 (C-13b), 109.20 (C-1), 108.38 (C-4), 102.08 (O-CH$_2$-O), 62.11 (C$_9$-OCH$_3$), 57.04 (C$_{10}$-OCH$_3$), 56.88 (C-6), 38.98 (1′-CH$_2$), 37.45 (-NH-CO-CH$_2$-C$_{13}$), 31.35 (7′-CH$_2$), 29.06, 29.05, 28.83, 28.74 (2′-, 4′-, 5′-, 6′-CH$_2$), 27.23 (C-5), 26.52 (3′-CH$_2$), 22.13 (8′-CH$_2$), 13.99 (9′-CH$_3$).

Eukaryotic In Vitro Translation Inhibition Assay

Inhibition of the synthesis of firefly luciferase in the cell-free eukaryotic translation system by the studied compounds was tested in a lysate of HEK293T cells. The reaction was carried out in a total volume of 10 µL with the following reagents: 5 µL of HEK293T cell lysate

(S10 extract); 1 µL Translation Buffer 10x (200 mM HEPES-KOH pH 7.6, 10 mM dithiothreitol, 5 mM spermidine-HCl, 80 mM creatine phosphate, 10 mM ATP, 2 mM GTP, 0.25 mM of each amino acid); 0.4 µL of potassium acetate (2 mM); 1 µL of magnesium acetate (10 mM); 0.5 µL of D-luciferin (10 mM); 0.1 µL of RiboLock RNase Inhibitor (40 U/µL) (Thermo Fisher Scientific, Waltham, MA, USA); 1 µL of the tested compound or nuclease-free water; 1 µL of mRNA *Fluc* (50 ng), capped and with poly-A sequence. Reactions containing all components, except mRNA, were preincubated for 5 min with the tested compounds at a final concentration ranging from 5 to 1000 µM or with water. Then mRNA was added and the in vitro translation reaction was carried out at 37 °C for 1 h. The activity of in vitro synthesized luciferase was measured by VICTOR X5 Multilabel Plate Reader (PerkinElmer, Waltham, MA, USA) every 30 s.

II. SUPPLEMENTARY TABLE

Table S1. Bacterial strains used in the work.

Strain	Abbreviation
**Strains of *Escherichia coli***	
Escherichia coli JW5503 (*ΔtolC*) (KanS) pDualrep2	*E. coli ΔtolC* pDualrep2
Escherichia coli JW5503 (*ΔtolC*) (KanS)	*E. coli ΔtolC*
Escherichia coli JW5503 (*ΔtolC)* (KanS) pCA24N-*LacZ*	*E. coli ΔtolC*-CAT
Escherichia coli SQ110 (*ΔtolC)* (A2058G)	*E. coli* SQ110 *ΔtolC* (A2058G)
Escherichia coli ATCC 25922	*E. coli* ATCC
Escherichia coli K-12	*E. coli* K-12
**Strains of *Bacillus subtilis***	
Bacillus subtilis 168	*B. subtilis*
Bacillus subtilis ATCC 6633	*B. subtilis* ATCC
Bacillus subtilis 168 pHT01	*B. subtilis*-CAT
**Other strains**	
Leuconostoc mesenteroides VKPM B-4177	*L. mesenteroides*
Staphylococcus aureus INA 00761 (MRSA)	*St. aureus*
Mycobacterium smegmatis VKPM Ac 1339	*Myc. smegmatis* VKPM Ac 1339
Mycobacterium smegmatis mc^2155	*Myc. smegmatis* mc^2155
**Fungi**	
Aspergillus niger INA 00760	*A. niger*
Saccharomyces cerevisiae RIA 259	*S. cerevisiae*

III. SUPPLEMENTARY FIGURES

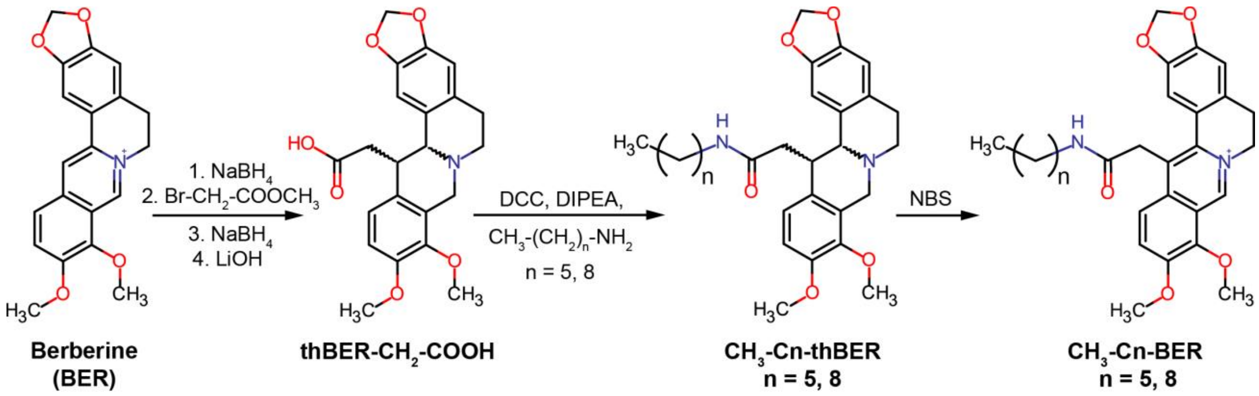

Figure S1. Scheme of the chemical synthesis of 13-alkyl-derivatives of berberine – CH_3-Cn-BER, n = 5, 8.

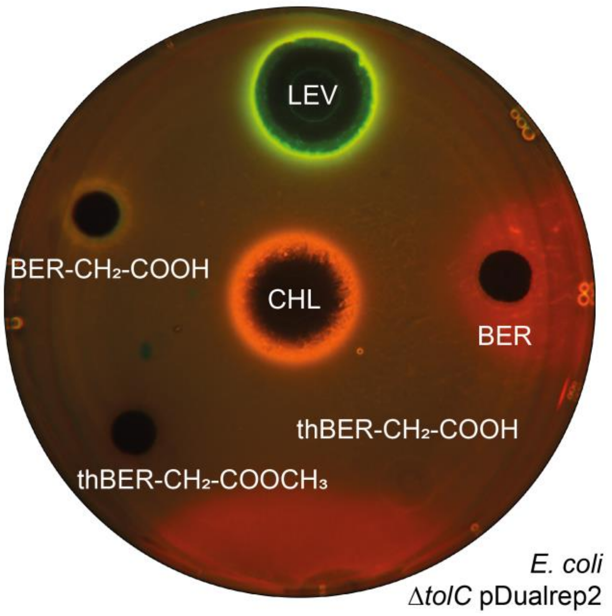

Figure S2. Testing of the BER, BER-CH_2-COOH, thBER-CH_2-$COOCH_3$, and thBER-CH_2-COOH antibacterial activity using *E. coli ΔtolC* pDualrep2 reporter strain. Levofloxacin (LEV) and chloramphenicol (CHL) are used as controls. The induction of the red fluorescent protein expression (green halo around the inhibition zone, pseudocolor) is triggered by DNA-damage, while the induction of Katushka2S protein (red halo, pseudocolor) occurs in response to ribosome stalling.

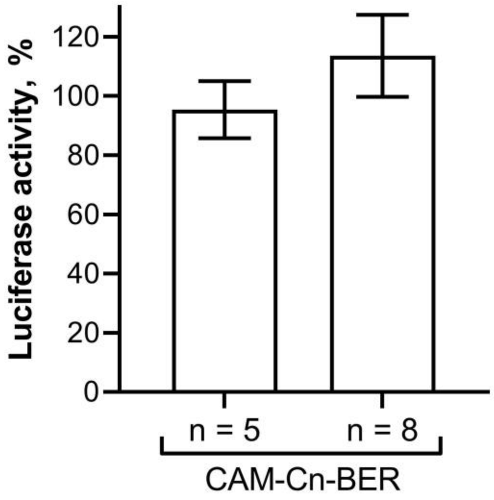

Figure S3. Eukaryotic translation. The inhibition of protein synthesis in vitro by 30 μM of CAM-C5-BER and CAM-C8-BER in the cell-free eukaryotic transcription-translation coupled system. The relative enzymatic activity of in vitro synthesized firefly luciferase is shown. The error-bars represent the standard deviations.

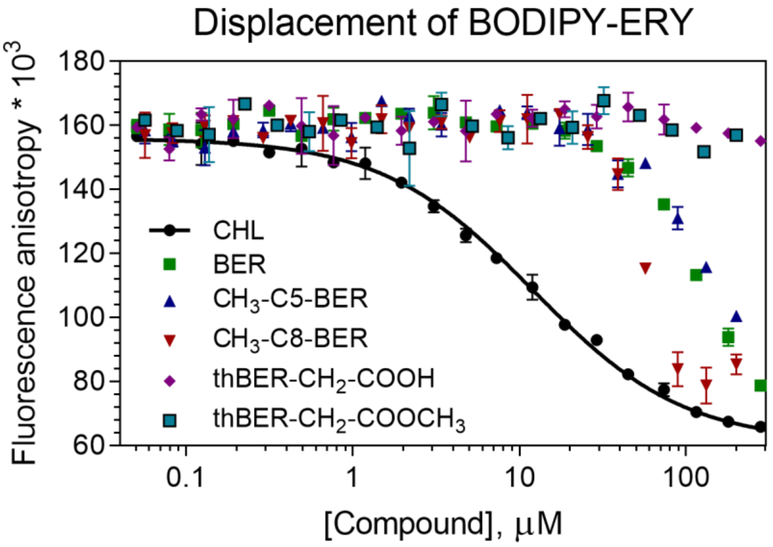

Figure S4. A competitive binding assay to test the affinity of CHL, BER, CH_3-Cn-BER (n = 5, 8), thBER-CH_2-COOH, and thBER-CH_2-$COOCH_3$ to *E. coli* 70S ribosomes measured by fluorescence anisotropy of fluorescently labeled analog of the erythromycin, BODIPY-ERY. All reactions were repeated at least two times. Error bars represent the standard deviation.

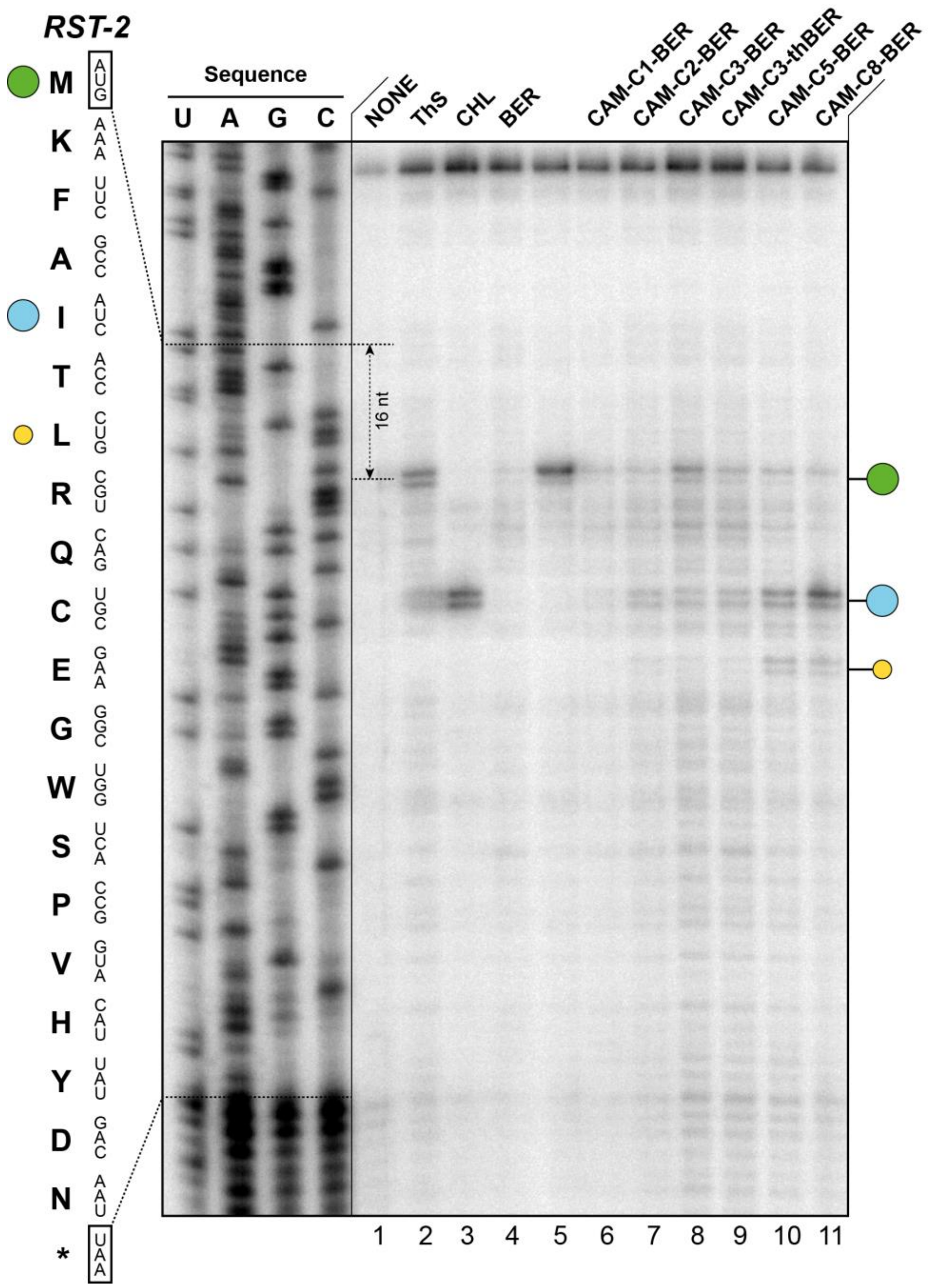

Figure S5. Original version of the Figure 3C. Ribosome stalling by CAM-Cn-BER (n = 1, 2, 3, 5, 8, lanes 6, 7, 8, 10, 11) and CAM-C3-thBER (lane 9) on *RST-2* mRNA as detected by a reverse-transcription primer-extension inhibition (toeprinting) assay in a cell-free translation system. DMSO, 0.5% (NONE, lane 1), ThS (inhibits translation at the start codon, lane 2), CHL (lane 3), and BER (lane 4) were used as controls. The nucleotide sequence of *RST-2* mRNA and its corresponding amino acid sequence are shown on the left. The green circle marks the translation arrest at the start codon, while the blue and yellow circles denote drug-induced arrest sites within the coding sequence of the mRNA used. Note that due to the large size of the ribosome, the reverse transcriptase used in the toeprinting assay stops 16 nucleotides downstream of the codon located in the P-site. The asterisk indicates a stop codon.

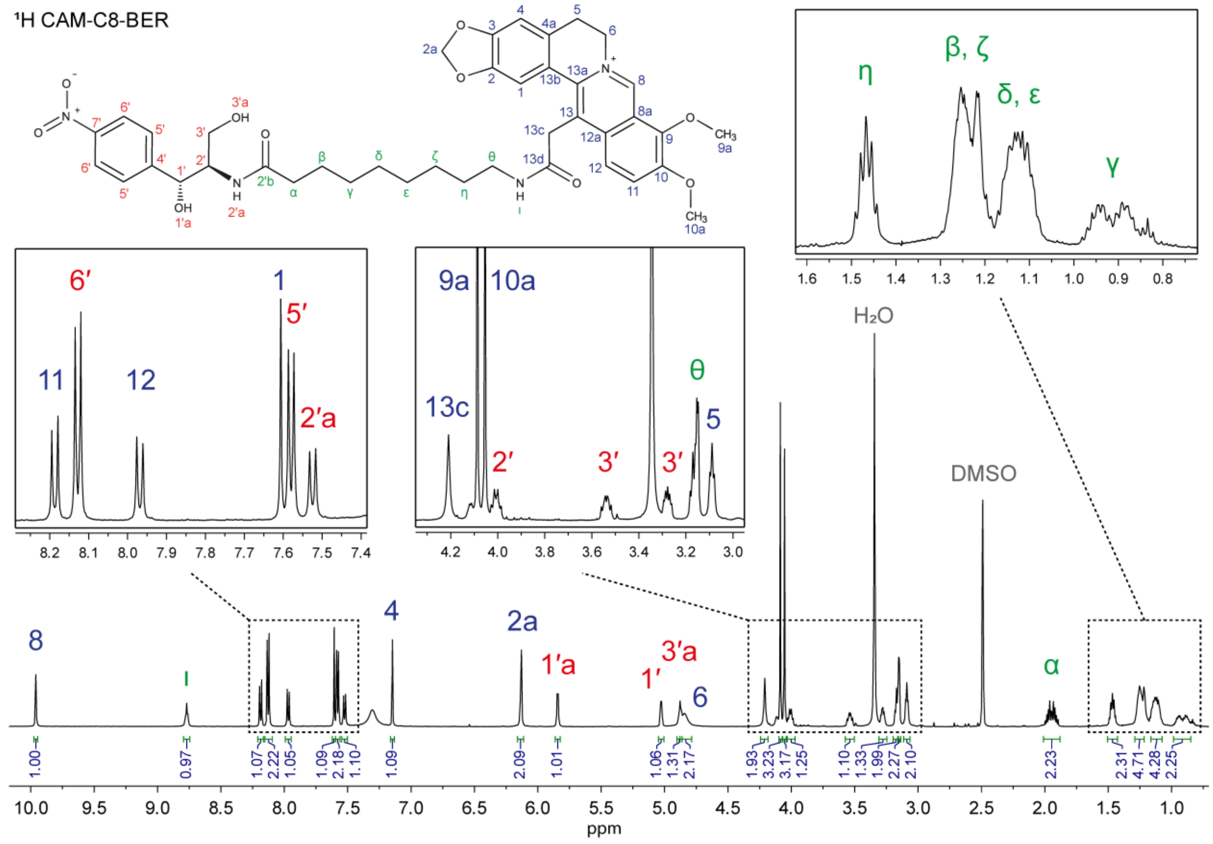

Figure S6. ^{1}H NMR spectrum of CAM-C8-BER.

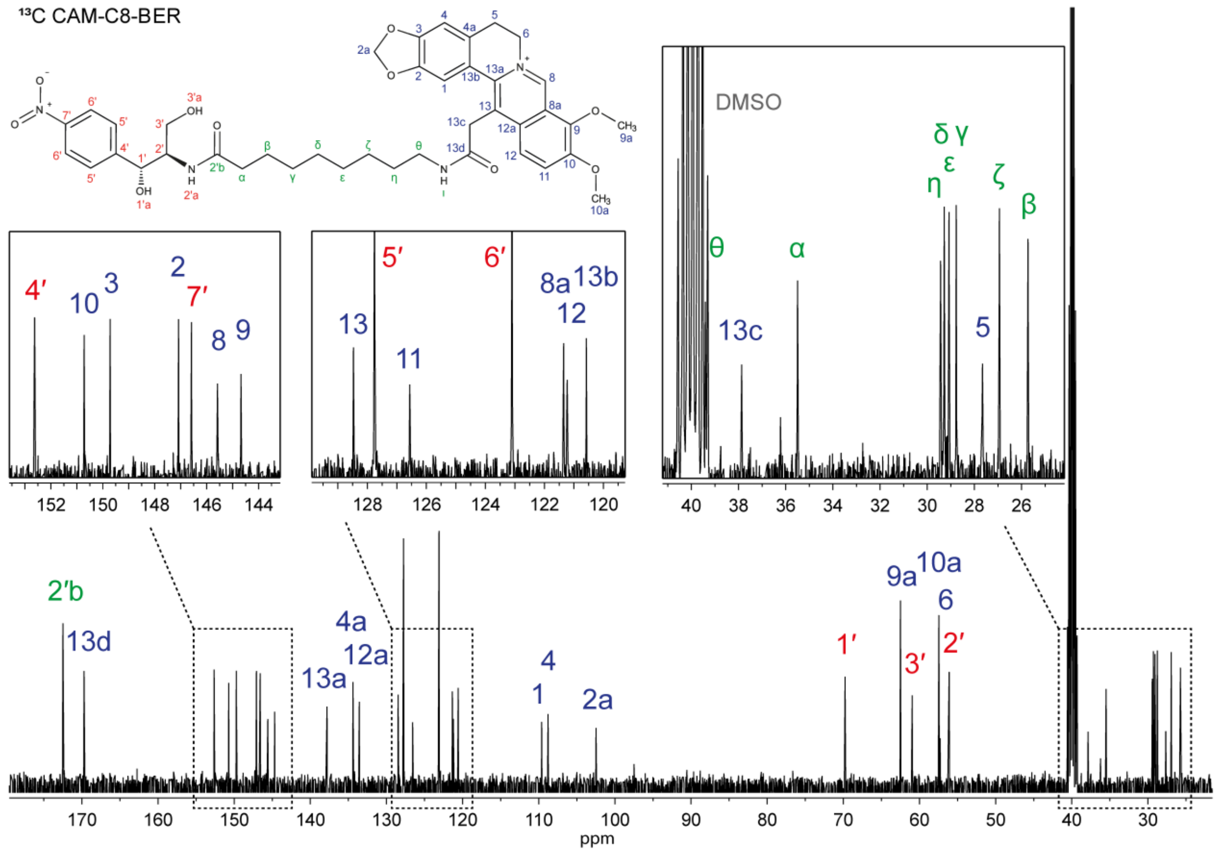

Figure S7. ^{13}C NMR spectrum of CAM-C8-BER.

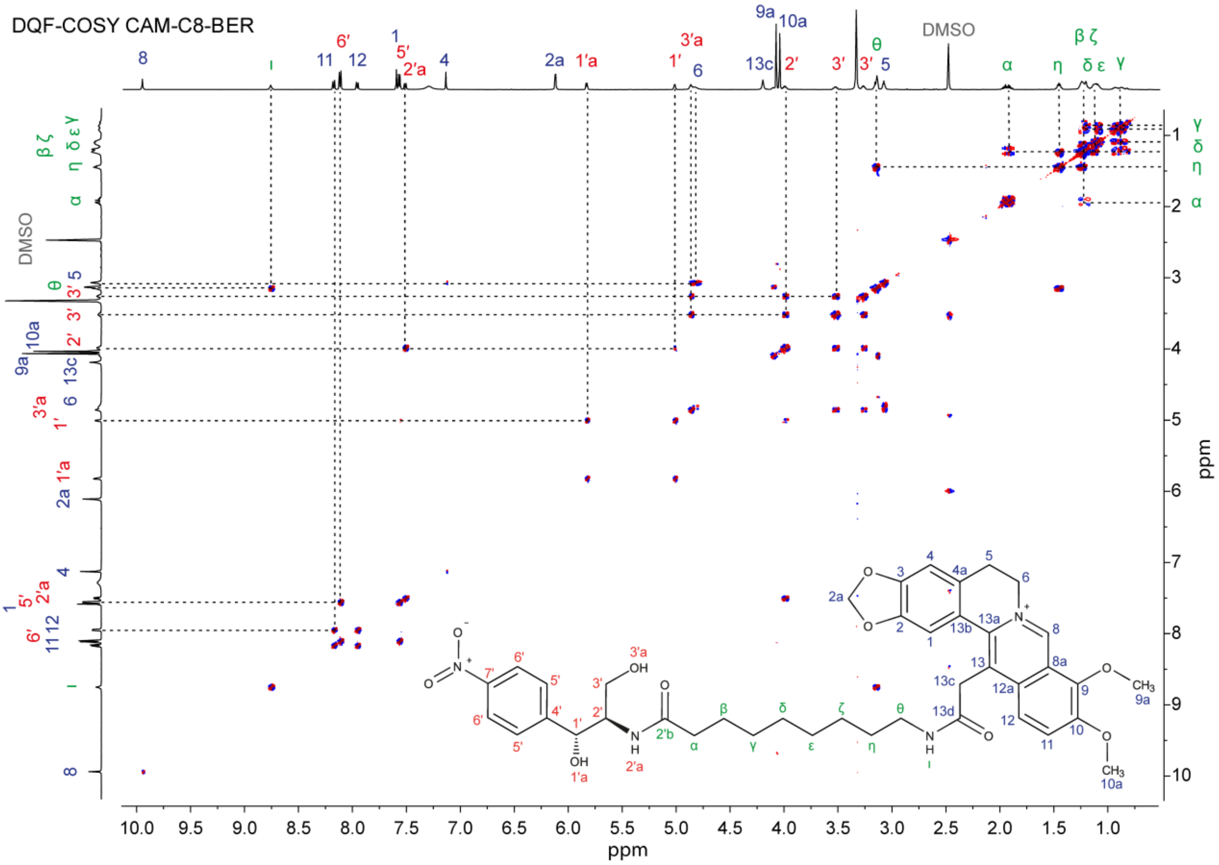

Figure S8. DQF-COSY NMR spectrum of CAM-C8-BER.

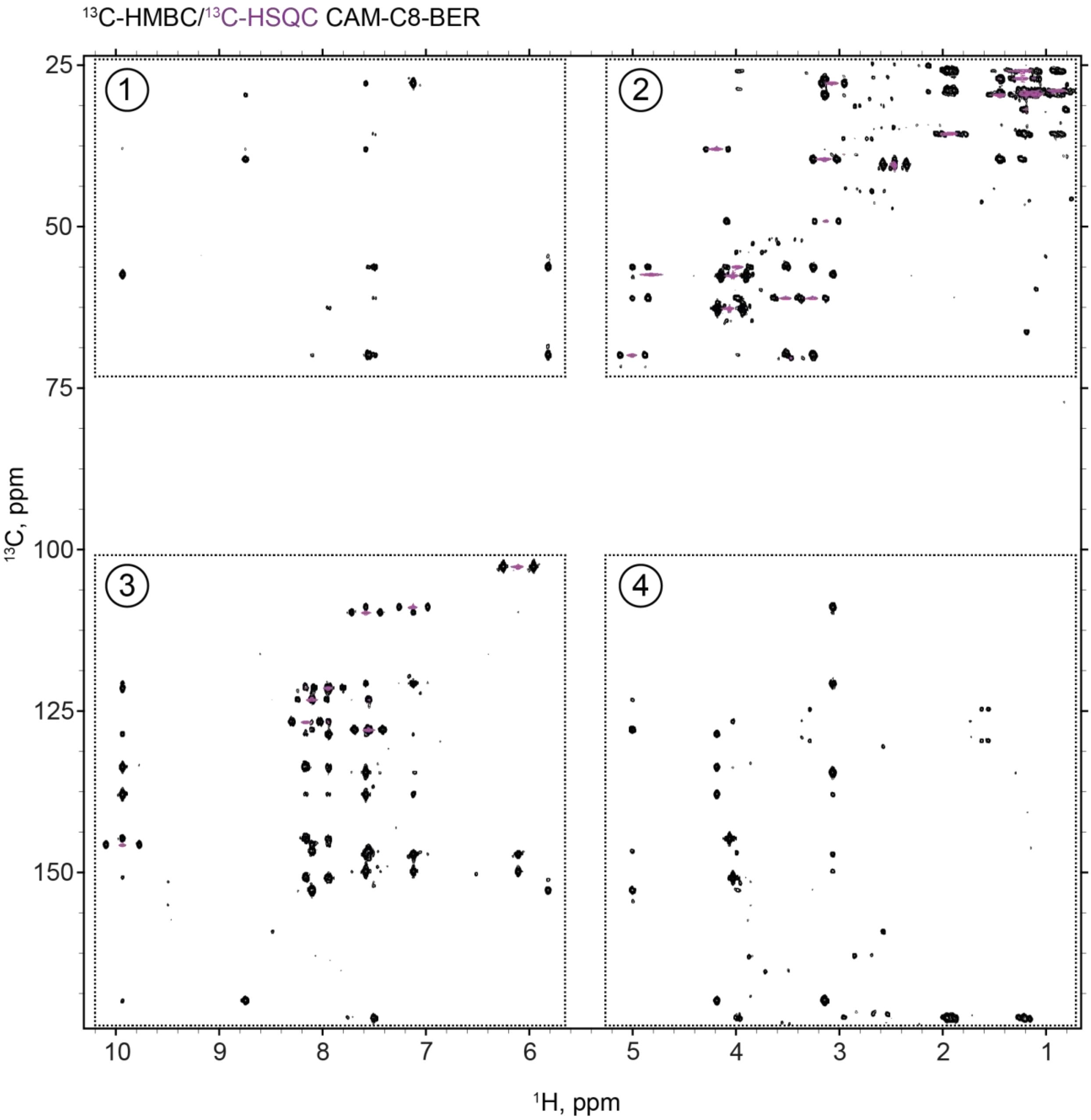

Figure S9. Overlay of ^{1}H-^{13}C HMBC (black) and ^{1}H-^{13}C HSQC (magenta) NMR spectra of CAM-C8-BER. The general view of spectra is shown.

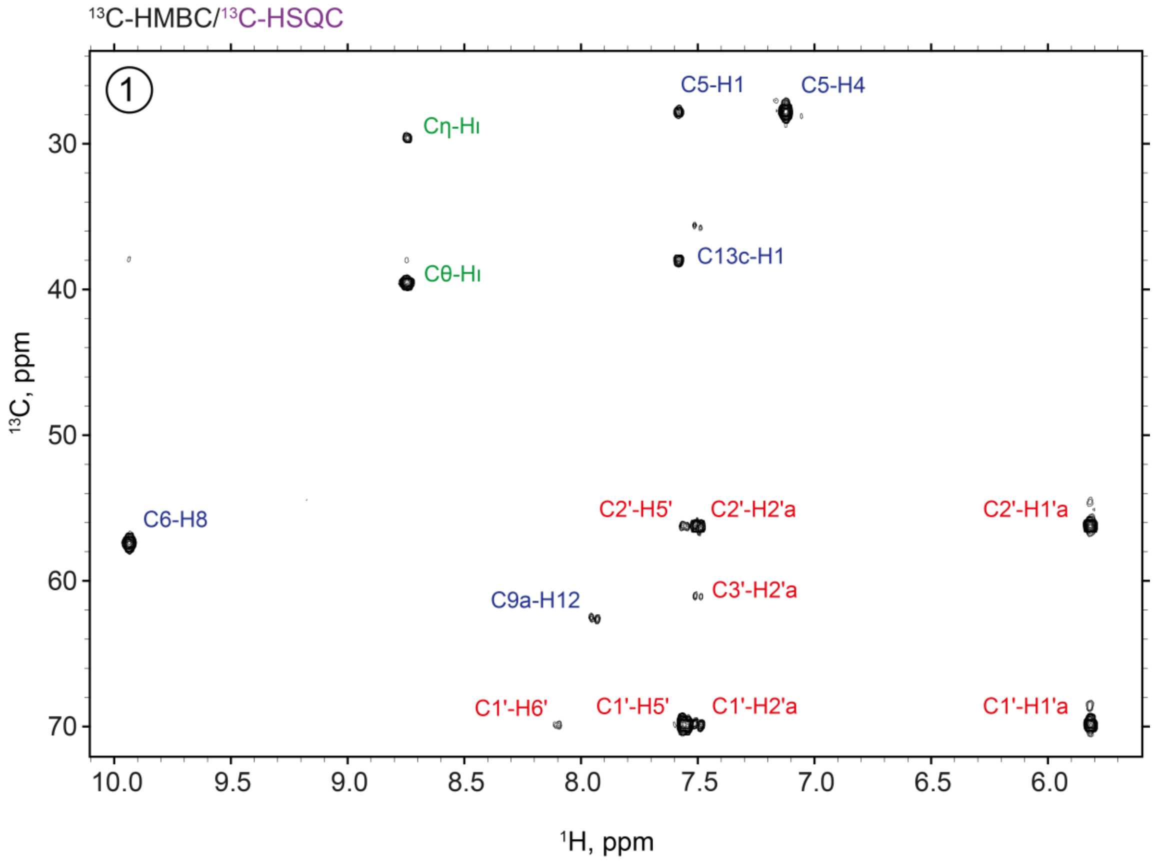

Figure S10. Overlay of ^{1}H-^{13}C HMBC (black) and ^{1}H-^{13}C HSQC (magenta) NMR spectra of CAM-C8-BER (area 1).

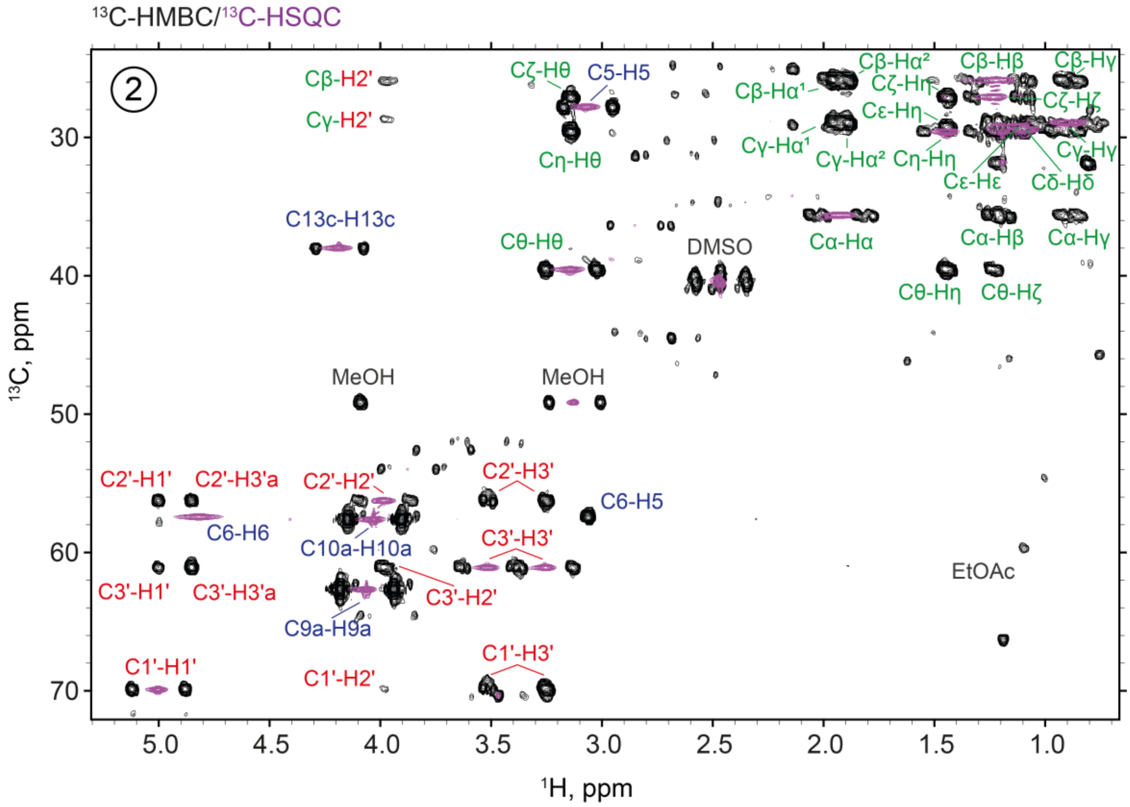

Figure S11. Overlay of ^{1}H-^{13}C HMBC (black) and ^{1}H-^{13}C HSQC (magenta) NMR spectra of CAM-C8-BER (area 2).

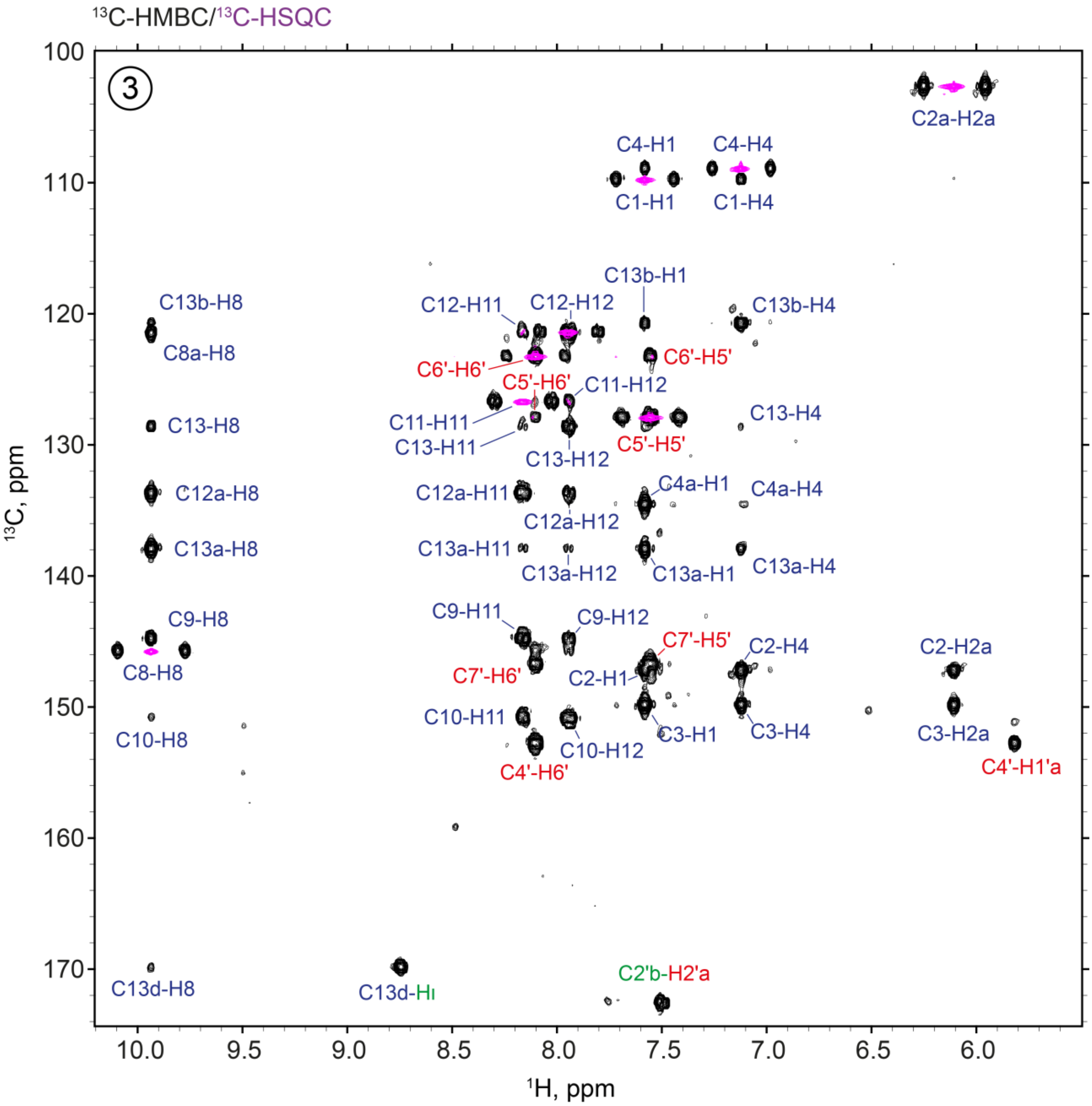

Figure S12. Overlay of ^{1}H-^{13}C HMBC (black) and ^{1}H-^{13}C HSQC (magenta) NMR spectra of CAM-C8-BER (area 3).

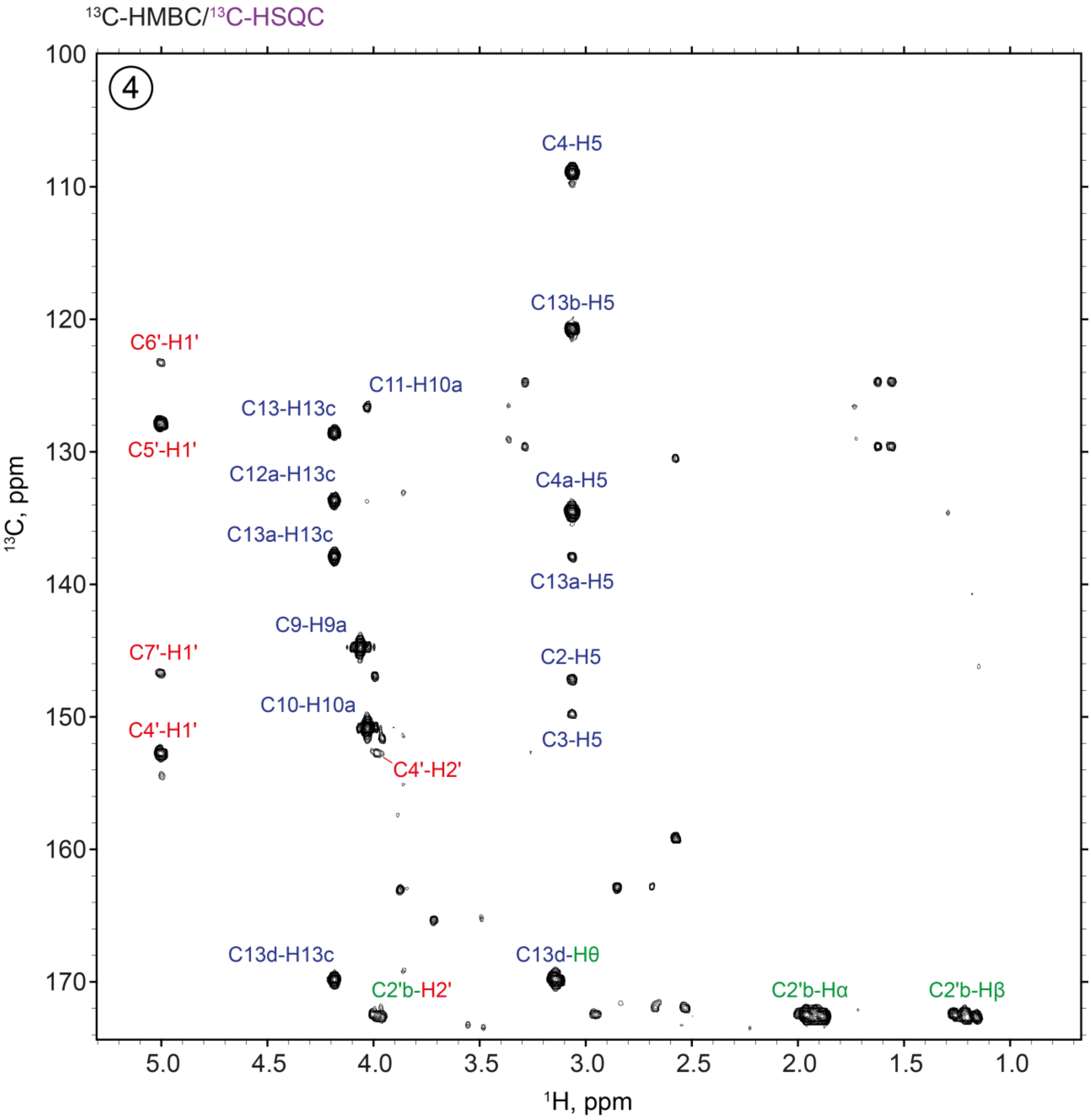

Figure S13. Overlay of ^{1}H-^{13}C HMBC (black) and ^{1}H-^{13}C HSQC (magenta) NMR spectra of CAM-C8-BER (area 4).

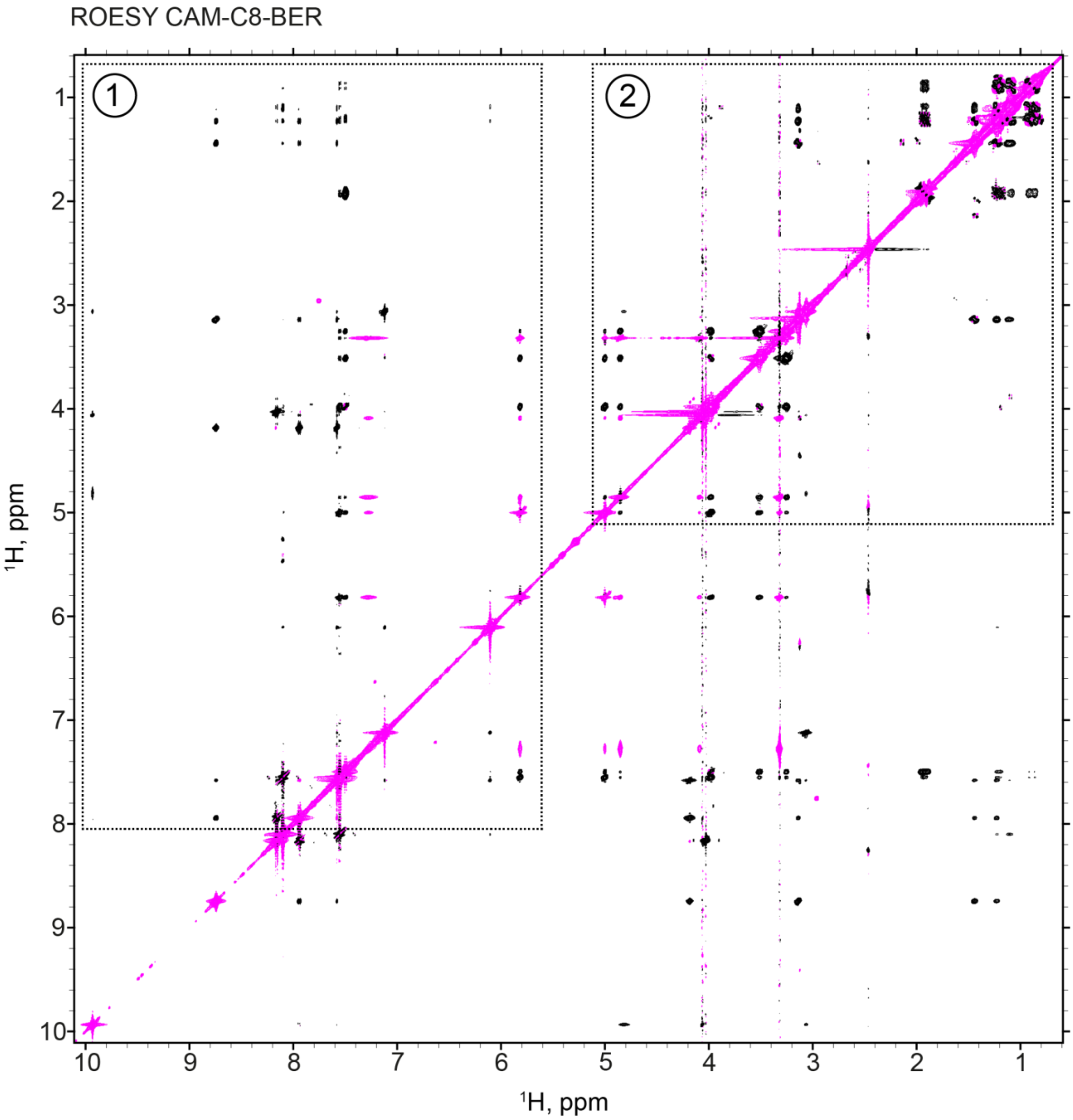

Figure S14. ^1H-^1H ROESY NMR spectrum of CAM-C8-BER. The general view of the spectrum is shown.

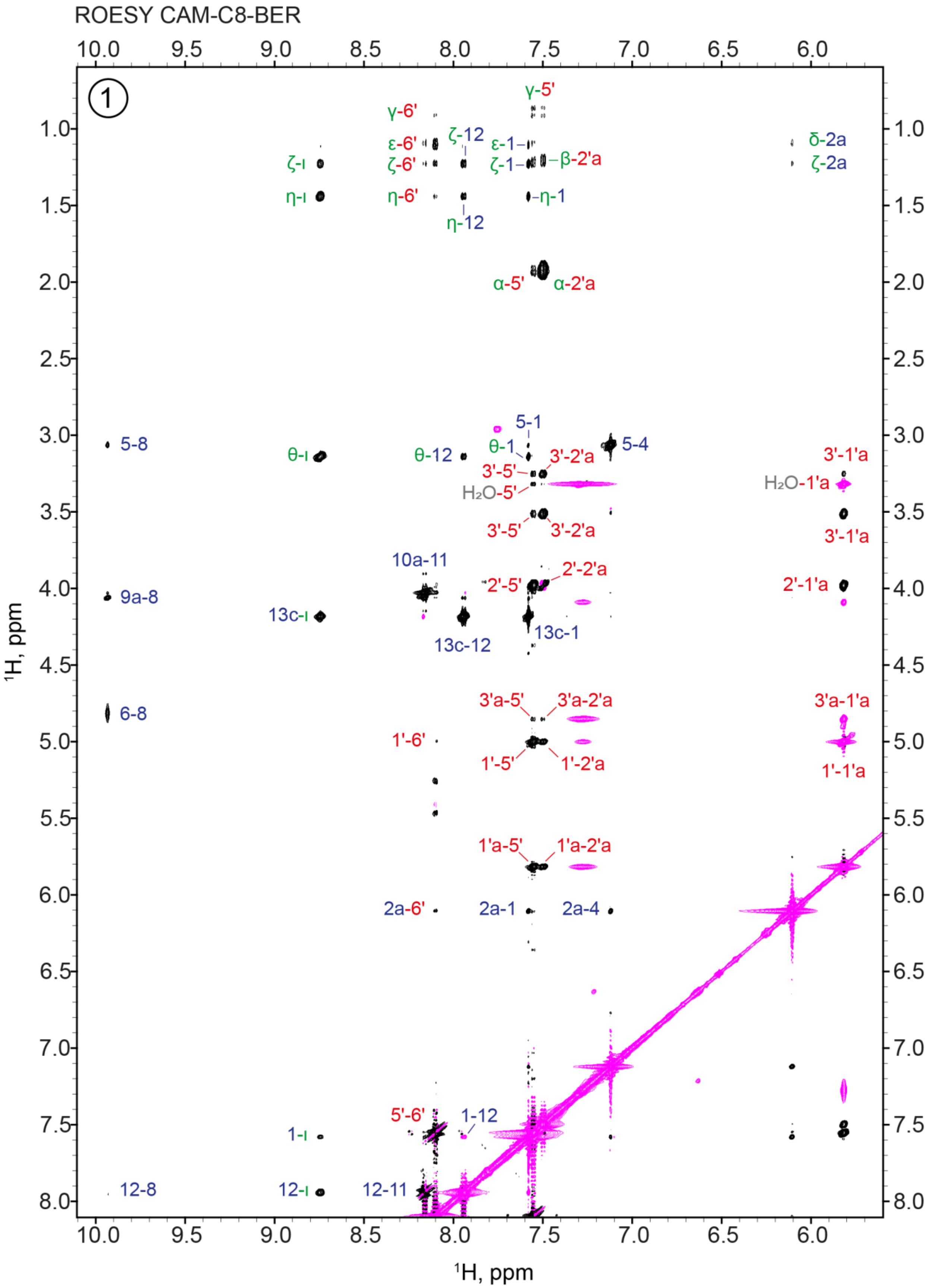

Figure S15. ^1H-^1H ROESY NMR spectrum of CAM-C8-BER (area 1).

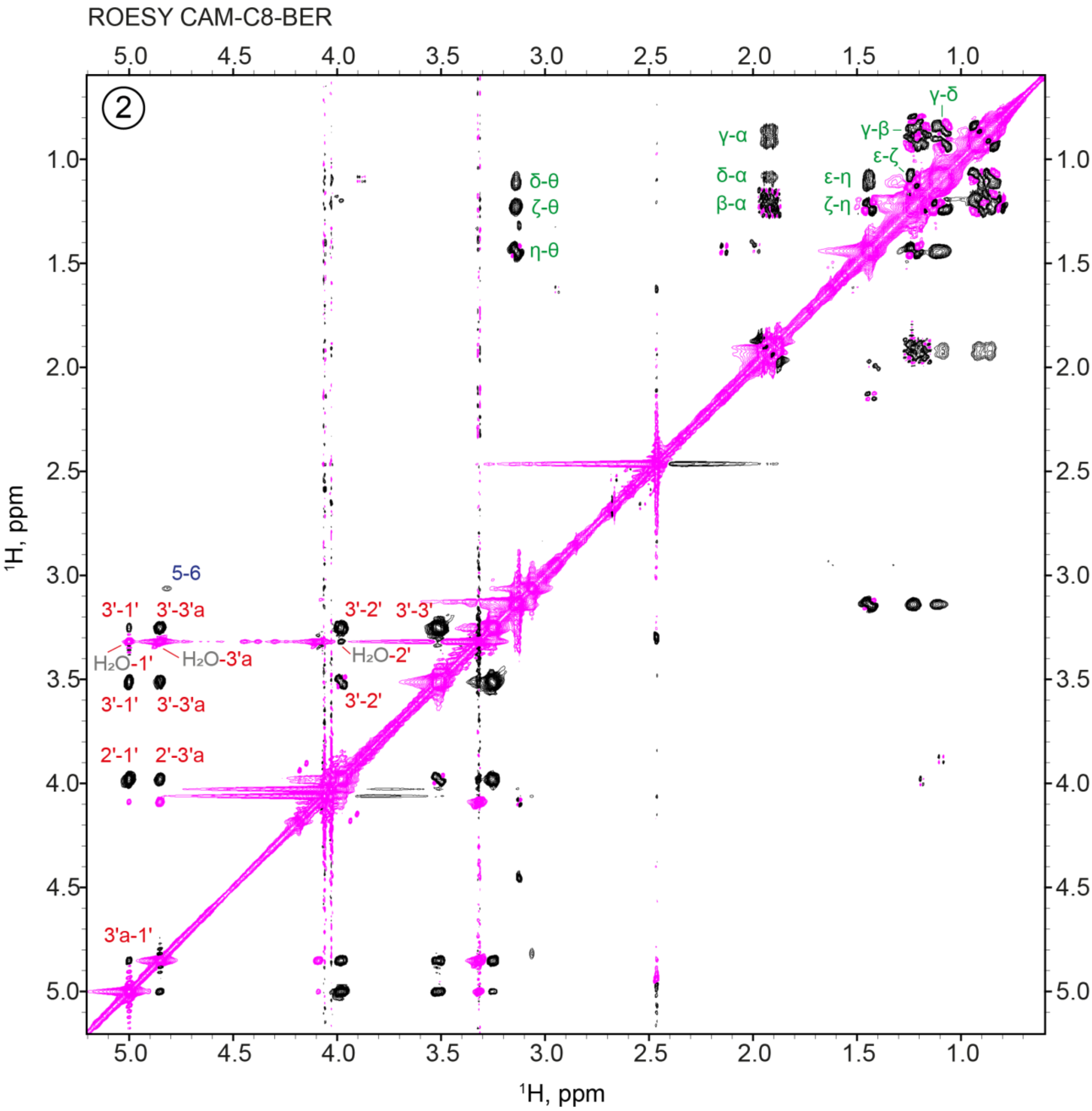

Figure S16. ^1H-^1H ROESY NMR spectrum of CAM-C8-BER (area 2).
